# Efficacy of platinum-based chemotherapy in metastatic breast cancer and HRD biomarkers: utility of exome sequencing

**DOI:** 10.1038/s41523-022-00395-0

**Published:** 2022-03-04

**Authors:** Loïck Galland, Elise Ballot, Hugo Mananet, Romain Boidot, Julie Lecuelle, Juliette Albuisson, Laurent Arnould, Isabelle Desmoulins, Didier Mayeur, Courèche Kaderbhai, Silvia Ilie, Audrey Hennequin, Anthony Bergeron, Valentin Derangère, François Ghiringhelli, Caroline Truntzer, Sylvain Ladoire

**Affiliations:** 1 Department of Medical Oncology, Centre Georges François Leclerc, Dijon, France; 2 Platform of Transfer in Biological Oncology, Georges François Leclerc Cancer Center, Dijon, France; 3 University of Burgundy-Franche Comté, Besançon, France; 4 Department of Pathology and Tumor Biology, Centre Georges François Leclerc, Dijon, France; 5 Genomic and Immunotherapy Medical Institute, Dijon University Hospital, Dijon, France; 6 Centre de Recherche INSERM LNC-UMR1231, Dijon, France

**Keywords:** Tumour biomarkers, Breast cancer, Predictive markers

## Abstract

Metastatic breast cancer (MBC) is frequently managed by platinum-based chemotherapy during the disease course. The real benefit of these treatments is uncertain at advanced stages of the disease and in non-triple-negative subtypes. Since homologous recombination deficiency (HRD) could inform about tumor sensitivity to DNA-damaging agents, we aimed to determine biomarkers of genomic instability, and their link with platinum efficacy. In this single-center study, we report *BRCA1/2* mutational status, HRD score and signature 3 levels, all obtained by tumor exome sequencing, in 86 patients with various subtypes of MBC and who received platinum-based chemotherapy. Overall response rate, disease control rate, PFS and PFS2/PFS1 ratio were evaluated to assess platinum-based chemotherapy efficacy. Among the 86 tumor samples analyzed, 7 harbored *BRCA1/2* mutations. We found a subset of *BRCA*-proficient MBC with high HRD score or high S3 levels, comparable to *BRCA*-mutated tumors. However, these patients with high HRD score or high S3 tumor level do not seem to benefit more from platinum-based chemotherapy than the others, in terms of response rates and/or PFS, regardless of BC molecular subtype. By multivariate analysis, only the absence of liver metastases was independently associated with significantly better PFS on platinum-based chemotherapy. However, some of our exploratory analyses reveal that certain methods, when optimized, seem to associate with platinum benefit. Tumor exome sequencing methodology for quantifying HRD has to be approached systematically, and further validated and standardized prior to its clinical use. Further studies are warranted to confirm these results to guide platinum use in MBC.

## Introduction

Breast cancer is the most common malignancy among women worldwide^1^. In metastatic breast cancer (MBC), therapy goals are prolongation of survival and maintaining the quality of life. Chemotherapy is one of the most widely used systemic therapies, and many families of molecules have shown their effectiveness in MBC, even in very advanced lines of treatment. Besides mitotic spindle poisons (taxanes, eribulin), anthracyclines, or oral fluoropyrimidines, platinum salts (cisplatin or carboplatin in monotherapy or associated with gemcitabine) have shown high response rates in several studies in patients with MBC, especially in triple-negative (TNBC) subtypes, in which homologous recombination deficiency (HRD) is more frequent^2^. Indeed, tumors with HRD have an impaired ability to repair double-strand DNA breaks, and could therefore be more sensitive to PARP inhibitors^3^ or DNA-damaging chemotherapy agents, such as platinum salts^4^.

For example, the addition of platinum to standard neoadjuvant chemotherapy results in greater pathological complete response (pCR) rates in patients with localized TNBC^5,6^. About 15–20% of TNBC occur in the context of germline mutation of *BRCA1* or *BRCA2 (gBRCA1/2)*, which are key genes involved in the homologous recombination (HR) process^7^. Thus, pCR rates appear to be very high, ranging from 20 to 60%, in *BRCA*-mutated TNBC patients treated with cisplatin monotherapy^8,9^.

In the metastatic setting, the triple-negative breast cancer trial (TNT)^10^ randomly assigned patients with metastatic TNBC to either docetaxel or carboplatin in the first line of treatment. Results showed that carboplatin was associated with a significantly higher overall response rate (ORR) and progression free-survival (PFS) for the 43 germline-BRCA mutation carriers enrolled, in contrast to those without *BRCA* mutation. Importantly, beyond *BRCA1/2* mutations, many other genomic and epigenetic alterations may explain inactivation of different HR components, leading to HRD in *BRCA* proficient tumors (whether it is a TNBC subtype or not)^11–13^.

Given the uncertain benefit of platinum-based chemotherapies in non-TNBC, and/or in *BRCA* proficient MBC^14^, it appears important to know whether the degree of HRD is associated with the benefit of such DNA-damaging agents, in order to guide the choice of chemotherapy.

Analysis of mutational signatures associated with HRD such as “signature 3” (S3)^15–17^, or signatures designed to capture “genomic scars” associated with HRD, regardless of etiological mechanism, are commonly used to identify tumors that may benefit from platinum or PARP inhibitor treatment in ovarian cancer^18^. Among these genomic scar signatures, the “myChoice HRD” test defined HRD-positive tumors based on an HRD score ≥42^19^. These assays may use next-generation sequencing (NGS) performed on formalin-fixed paraffin-embedded (FFPE) tumor samples^20–22^.

Thus, in this single-center study using tumor exome analysis from 86 MBC patients (all subtypes) and treated with platinum-based chemotherapy in the metastatic setting, we aimed to determine genomic instability (assessed by HRD and S3 scores, or point mutations within the genes involved in HR), and their link with platinum efficacy.

Within this work, we will interchangeably use “BRCA-WT” or “BRCA proficient” term to design patients without BRCA1/2 observed mutations, whether from germline or tumoral origin.

## Results

### Patients’ clinical characteristics

Between June 2018 and March 2020, 86 women with a histology-confirmed MBC were enrolled. The characteristics of the 86 patients are detailed in Table 1. Briefly, 75 patients (87%) had metastatic ductal carcinoma, and 9 (10%) had metastatic lobular carcinoma. Thirty patients (35%) had triple-negative breast cancer, which was defined as oestogren and progesterone receptor levels of less than 1%, and HER2-negative. Thirteen patients had HER2-positive tumors (15%), and 43 had HER2-negative and ER-positive tumors (50%). A majority of these patients (57%) had polymetastatic disease, with ≥ 4 different metastatic sites involved. Eighty-one (94%) patients had visceral metastasis at the time of platinum use. Among metastatic sites, 65% of patients harbored liver metastases, 74% had bone metastases, and 24% had cerebral metastases history.

**Table 1:** Baseline characteristics of the study patients (*N* = 86).

	*N* (%)
*Age class at diagnosis of MBC*	
≤50 years	48 (56%)
>50 years	38 (44%)
*WHO performance status*	
0	23 (27%)
1	34 (40%)
2	17 (20%)
3	5 (6%)
Histology	
Ductal	75 (87%)
Lobular	9 (10%)
Others	3 (3%)
*Breast cancer histological subtypes*	
HER2 amplified	13 (15%)
ER+/HER−	43 (50%)
Triple negative	30 (35%)
*Prior adjuvant or neoadjuvant chemotherapy*	
No	29 (34%)
Yes	57 (66%)
*Previous systemic treatment exposure in metastatic setting*	
Endocrine therapy	19 (22%)
Endocrine therapy and targeted therapy	34 (40%)
Anthracycline	17 (20%)
Taxane (Paclitaxel or Docetaxel)	64 (74%)
Eribulin	29 (34%)
Capecitabine	53 (62%)
*Metastatic free interval*	
Median (years) [range]	2.46 [0; 24]
De novo	6 (7%)
Relapse	80 (93%)
*Number of metastasic sites*	
1	5 (6%)
2	16 (19%)
3	16 (19%)
≥4	49 (57%)
Visceral metastasis	81 (94%)
Liver & Lung	33 (38%)
Liver only	23 (27%)
Lung only	15 (17%)
Others	10 (12%)
*Bone metastasis*	
Bone metastasis only	1 (1%)
With other metastasis	85 (99%)
Cerebral metastasis	
No	65 (76%)
Yes	21 (24%)
*Platinum treatment line*	
Median	4
1	18 (21%)
2	9 (10%)
3	10 (12%)
4	12 (14%)
≥5	37 (43%)
*Platinum chemotherapy schedule*	
Carboplatin-based	86 (all)
Carboplatin and cisplatin-based (monotherapy)	2 (2%)
Cisplatin-based	0 (0)
Carboplatin + gemcitabine-based	58 (67%)
Carboplatin and Cisplatin + gemcitabine-based	8 (9%)
*BRCA mutation*	
No	79 (82%)
Yes	7 (8%)
Germline	4 (5%)
Somatic	3 (3%)

### Chemotherapy

All 86 patients received platinum-based chemotherapy (regardless of the line of treatment).

Platinum-based chemotherapy was associated with gemcitabine in 66 patients (77%). Ten patients initially received cisplatin, before a rapid switch to carboplatin because of adverse effects. Platinum-based chemotherapy was used in first-line treatment for 18 patients (21%), and in second line or more for 68 patients (79%). The median line of platinum-based chemotherapy was 4 months [1; 18], and 53% of patients received this chemotherapy in 5th line or more. Patients with *BRCA1/2* mutated tumors tended to have received platinum earlier in the disease course, without the difference being significant when compared with patients with *BRCA1/2 WT* tumor status (*p* = 0.12). Carboplatin was mainly administered in an every 3 weeks schedule (*n* = 35 patients; 41%) (mean AUC: 4.89), or a weekly schedule (*n* = 46; 53.5%) (mean AUC: 2). The median number of lines of treatment (including other chemotherapies, and endocrine-based treatment) before platinum salts was 5 (range: 1–9). Capecitabine (62%), taxanes (74%), and eribulin (34%) were the three most frequent drugs used before platinum-based chemotherapy. Only 20% received anthracyclines prior to platinum. No patient received platinum or PARP inhibitor therapy in a (neo)adjuvant or metastatic setting, before enrollment in this cohort.

### Homologous recombination biomarker tumor status

Seven (8%) patients were *BRCA 1/2* mutated carriers (5 with germline mutation and 2 with somatic mutation; 5 with biallelic inactivation). Three of these patients had ER-positive disease and 4 had triple-negative breast cancer. None had HER2-positive MBC (Supplementary Table 1). On the assessment of pathogenic gene variations involved in HR other than *BRCA 1/2* mutations, 2 patients had frameshift variant on *ATM*, and one on *ATR* (Supplementary Table 1). The mean HRD score in the whole cohort was 27, and the median score was 26.25. Using the classical cutoff value of 42, high-level HRD score was found in 11 *BRCA 1/2 WT* patients (13%) and in 4 *BRCA 1/2* mutated tumors (5%).

We performed a comparison of our cohort with early breast cancer (eBC) in The Cancer Genome Atlas (TCGA): No significant difference in HRD score was found between early *BRCA* mutated tumors from TCGA, and metastatic *BRCA* mutated tumor from our cohort, since S3 level was slightly higher in ER+/HER2− patients in our cohort than in TCGA. In *BRCA WT* tumors, luminal tumors in our cohort of metastatic patients had a higher HRD score and S3 level than eBC TCGA tumors, which was not the case for TNBC or HER2-positive tumors (Supplementary Fig. 1a–d).

**Fig. 1:**
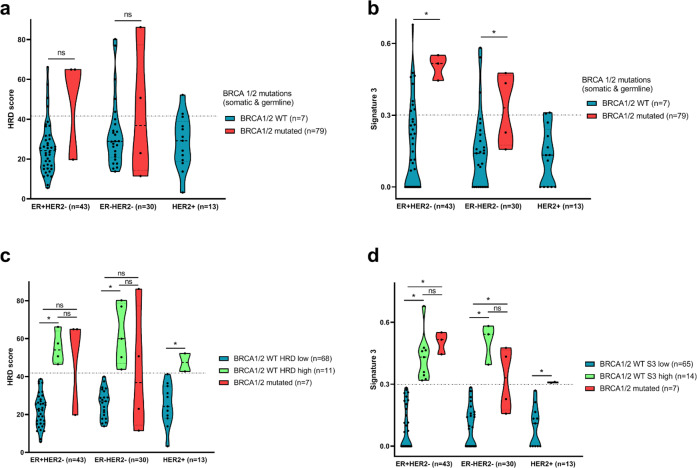
Distribution of HRD score and S3 levels according to BRCA 1/2 mutational status and breast cancermolecular subtypes. **a**, **b** Violin plots representing the distribution of HRD score (**a**) and signature 3 (**b**) according to molecular subtype and BRCA 1/2 mutational status. *: significant Wilcoxon test *p*-value. **c**, **d** Violin plots representing the distribution of HRD score (**c**) and signature 3 (**d**) according to molecular subtype and BRCA 1/2 mutational status combined with HRD status. Patients were stratified into three groups for each molecular subtype: BRCA 1/2 mutated tumors (red), BRCA1/2 wild type with high-HRD score (or high-S3 level) tumors (green), and BRCA 1/2 wild- type with low-HRD score (or low-S3 level) tumors (blue). *: significant Wilcoxon test *p*-value.

In our cohort, no significant association was found between the median HRD score and *BRCA* mutational status (despite a clear trend towards higher HRD scores in *BRCA 1/2* mutated tumors: respectively, 50.6 for patients with *BRCA 1/2* mutation and 26.3 for patients with *WT* status, *p*-value = 0.17) in the whole cohort, or in each BC subtype (Fig. 1a).

High levels of S3 were found in 14 (16%) *BRCA 1/2 WT* tumors and in 5 *BRCA 1/2* mutated tumors. Higher levels of S3 were found in *BRCA* mutated tumors (*p* = 0.01), both in ER+/HER2− and in triple-negative subtypes (Fig. 1b).

Of note, as other mutational signatures such as signature 8 have also been associated with HRD in breast cancer, we attempted to evaluate them in our series. Since only 2 tumors out of the 86 had signature 8, no further analyses were performed.

### Subgroups of HRD-high and S3-high tumors among *BRCA WT* MBC

We next investigated whether, within each subtype of breast cancer, there were non-mutated *BRCA* tumors that had a high HRD score and/or a high level of S3. Thus, considering our cohort, we then described and compared the three different groups of tumors, according to previously described cutoff values: *BRCA1/2* mutated, *BRCA WT* HRD-high and *BRCA WT* HRD-low (and *BRCA1/2* mutated, *BRCA WT* S3-high and *BRCA WT* S3-low).

Concerning HRD score, median HRD score was 52.2 in patients with HRD-high and 24 in patients with HRD-low status (*p*-value < 0.001). As expected, TAI, LOH and LST scores were significantly higher for HRD-high tumors (p-value < 0.001) (Supplementary Fig. 2a–c). Interestingly, the levels of HRD score in *BRCA WT* HRD-high tumors did not statistically significantly differ from *BRCA* mutated tumors, whatever the BC subtype (Fig. 1c). Similar results were observed concerning S3 levels, with subgroups of patients who had *BRCA WT* and S3-high tumor status in each BC subgroup (Fig. 1d).

Taken together, these results indicate that a small subset of *BRCA* proficient MBC actually has genomic features associated with HR, at comparable levels to those observed in their *BRCA* mutated counterparts.

### Association between genomic features quantifying tumor HRD and response to platinum-based chemotherapy

**Fig. 2:**
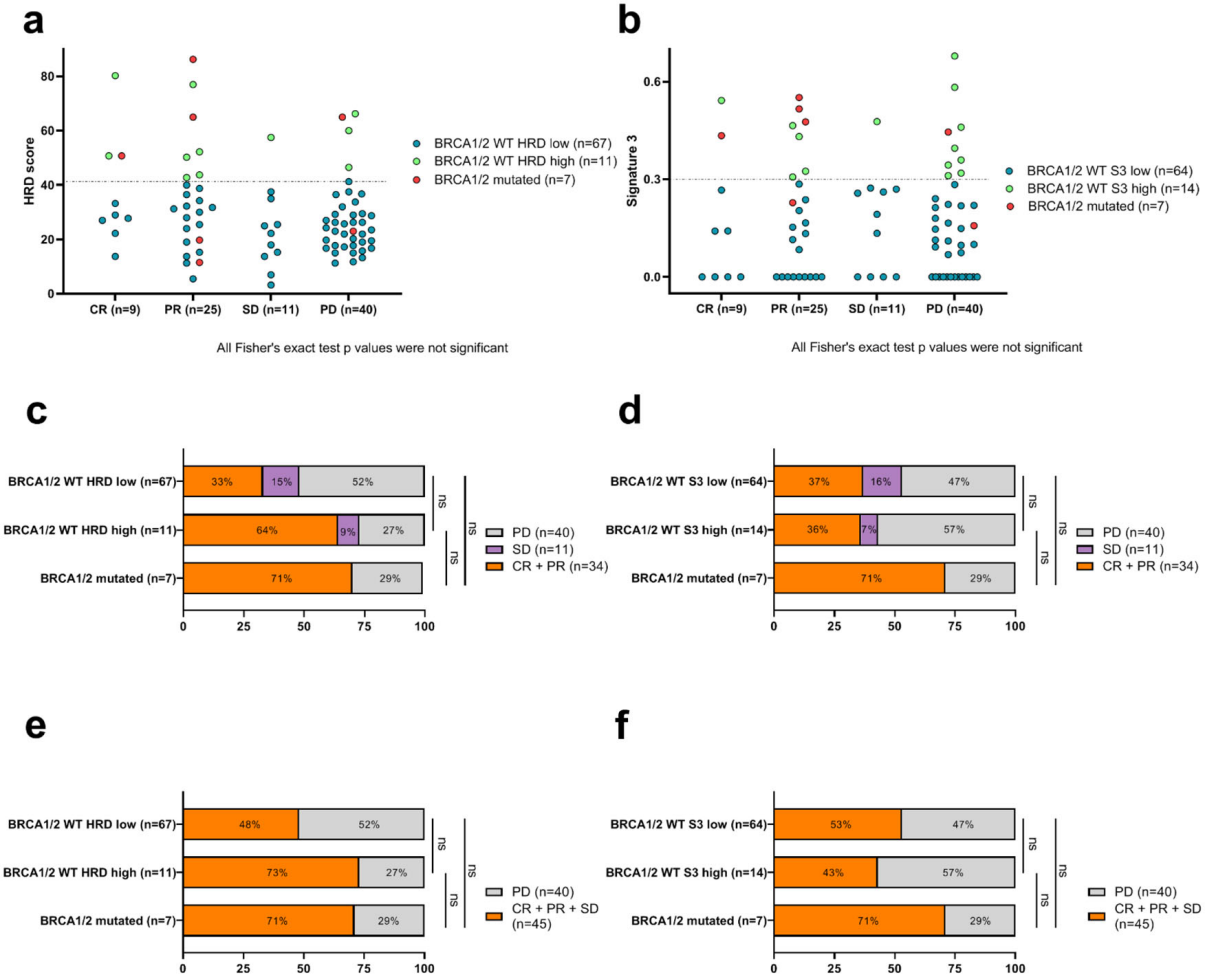
Association between HRD biomarkers and response to platinum-basedchemotherapy. **a**, **b** Dot plots representing the distribution of HRD score (**a**) and signature 3 level (**b**) according to response to platinum-based chemotherapy (CR: complete response; PR: partial response, SD: stable disease; PD: progressive disease). Points represent patients and colors identify the group formed with BRCA1/2 mutational and (**a**) HRD status or (**b**) signature 3 level. **c**, **d** Cumulative bar plots showing ORR: proportions of patients with complete response (CR) + partial response (PR), or stable disease (SD) or progressive disease (PD) are represented in each group formed with BRCA 1/2 mutated and (**c**) HRD status, or (**d**) signature 3 level. *: significant Fisher’sexact test *p*-value. **e**, **f** Cumulative bar plots showing DCR: proportions of patients with complete response (CR) + partial response (PR) + stable disease (SD), or progressive disease (PD) are represented in each group formed with BRCA 1/2 mutated and (**e**) HRD status or (**f**) signature 3 level. *: significant Fisher’sexact test *p*-value.

At the first radiological assessment under platinum therapy, 40 patients (47%) were considered to have PD. Among patients with non-PD, 9 (10%) had CR, 25 (29%) had PR, and 11 (13%) only had SD as the best response recorded. Response to platinum-based chemotherapy is presented according to HRD score in Fig. 2a and according to S3 in Fig. 2b. The proportion of patients with *BRCA* mutated, *BRCA WT* HRD-high, and *BRCA WT* HRD-low was not statistically significantly different among recorded response subgroups (namely, patients with CR, PR, SD, or PD) (Fig. 2a and Supplementary Fig. 3a). Similar results were observed when considering *BRCA WT* S3-high, and *BRCA WT* S3-low tumors (Fig. 2b and Supplementary Fig. 3b).

Considering ORR according to HRD score, there was a trend towards higher ORR in patients with *BRCA* mutated and patients with *BRCA WT* HRD-high tumors (ORR: 71% and 64%, respectively), compared to patients with *BRCA WT* HRD-low (ORR: 33%). However, these differences did not reach statistical significance: *p* = 1 (for *BRCA* mutated vs *BRCA WT* HRD-high), *p* = 0.15 (for *BRCA*-mutated vs *BRCA WT* HRD-low and for the comparison between *WT* HRD-high and *WT* HRD-low) (Fig. 2c). Additional analyses with exploratory cutoff values showed similar results with the cutoff value of 33, but interestingly, a statistically significantly better ORR was observed for patients with *BRCA* mutated and *BRCA-WT* HRD-high tumors, as compared to BRCA-WT HRD-low tumors, when the median HRD score of our cohort (26.25) was used at the cutoff (Supplementary Fig. 4a).

Considering tumors according to their S3 classification, despite a numerically higher ORR in patients with *BRCA* mutated tumor, there was no statistically significant difference between *BRCA* mutated and WT tumors, or between *BRCA WT* S3-high and *BRCA WT* S3-low tumors (*p* = 0.44, for *BRCA* mutated vs *BRCA WT* S3-high; *p* = 0.29, for *BRCA* mutated versus *WT* HRD-low; and *p* = 0.78, for *WT* HRD-high and *WT* HRD-low) (Fig. 2d). Non-significant results were also obtained using the median value of S3 (Supplementary Fig. 4b).

Similar non-statistically significant differences were observed when comparing the DCR according to tumor groups (Fig. 2e, f), despite a trend towards better DCR in patients with *BRCA* mutated tumors, or the *BRCA WT* HRD-high tumor subgroup. Similar non-statistically significant trends were also observed when exploratory cutoff values of 33 or 26.25 were tested (Supplementary Fig. 4c, d).

Interestingly, similar non-significant differences were observed when considering breast cancer molecular subgroups (Supplementary Fig. 3c, d).

When using SigMA to dichotomize S3-positive and S3-negative tumors, no objective response was detected in patients with S3-negative BRCA-WT tumors. Accordingly, there was a statistically higher ORR and DCR with platinum-based chemotherapy in patients with S3-positive tumor according to SigMA **(**Supplementary Fig. 5a, c), which translated into better PFS (Supplementary Fig. 5e). However, these exploratory results must be interpreted with caution, because the number of patients classified as S3-negative was very low. Output metrics from SigMA analysis are presented in Supplementary Table 2. Of note, when using SignatureAnalyser, we did not observe any significant difference between S3-high or S3-low (above or below the median value of our cohort) tumor, in terms of ORR, DCR, or survival (Supplementary Fig. 5b, d, f).

Furthermore, as current evidence shows that DNA-damaging agents like PARP inhibitors give better clinical results when used early, we explored the efficacy of platinum in our cohort when it was administered in the first or second line (*N* = 27 patients, of whom 3 had BRCA mutations). With the caveat of the small number of patients concerned, no difference in terms of DCR or ORR was observed in this population of patients treated early, whatever the HRD status group (whatever the cutoff used: 42, 33, or median value of our cohort) (Supplementary Fig. 6a, b).

Similarly, due to neoadjuvant clinical data indicating that there is no clear advantage of carboplatin in addition to standard chemotherapy when this includes anthracyclines, we analyzed the benefit of platinum-based chemotherapy according to whether the patient received pre-treatment with anthracyclines or not: patients who had not previously received anthracyclines were mainly those who had received early platinum-based chemotherapy, and the results are therefore similar to those obtained in this latter group of patients, with a trend towards higher response rates (DCR or ORR) in patients not previously treated with anthracyclines, regardless of HRD status (BRCA mutated, BRCA WT and HRD high, or BRCA WT and HRD low).

### Association between genomic features quantifying tumor HRD and survival obtained with platinum-based chemotherapy

**Fig. 3:**
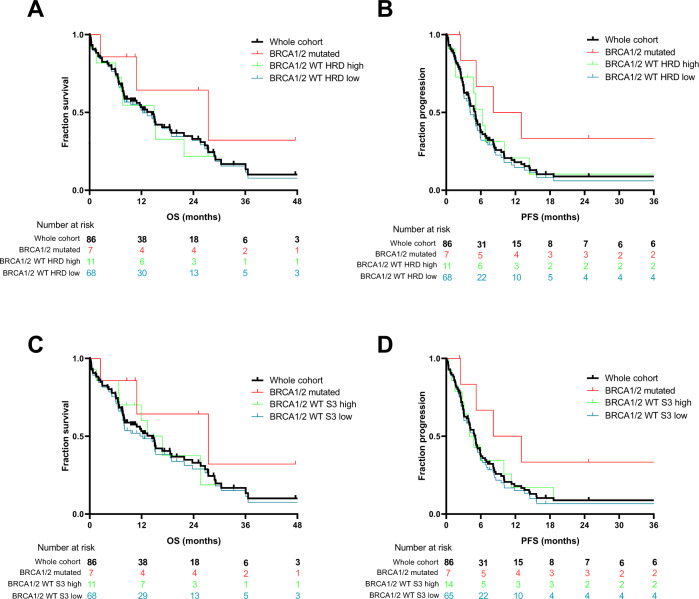
Progression free and overall survival for patients treated with platinum-basedchemotherapy. **A**, **B** Kaplan–Meier curve of overall survival (**A**) and progression free survival (**B**) for patients treated with platinum-based chemotherapy, according to BRCA 1/2 mutated and HRD score level. Black curves: whole cohort, red curves: patients with BRCA mutated tumors, green curves: patients with BRCA WT HRD-high tumors, blue curves: patients with BRCA WT HRD- low tumors. Ticks denote censored data. **C**, **D** Kaplan–Meier curve of overall survival (**C**) and progression free survival (**D**) for patients treated with platinum-based chemotherapy, according to BRCA 1/2 mutated and S3 level. Black curves: whole cohort, red curves: patients with BRCA mutated tumors, green curves: patients with BRCA WT S3-high tumors, blue curves: patients with BRCA WT S3-low tumors. Ticks denote censored data.

**Fig. 4:**
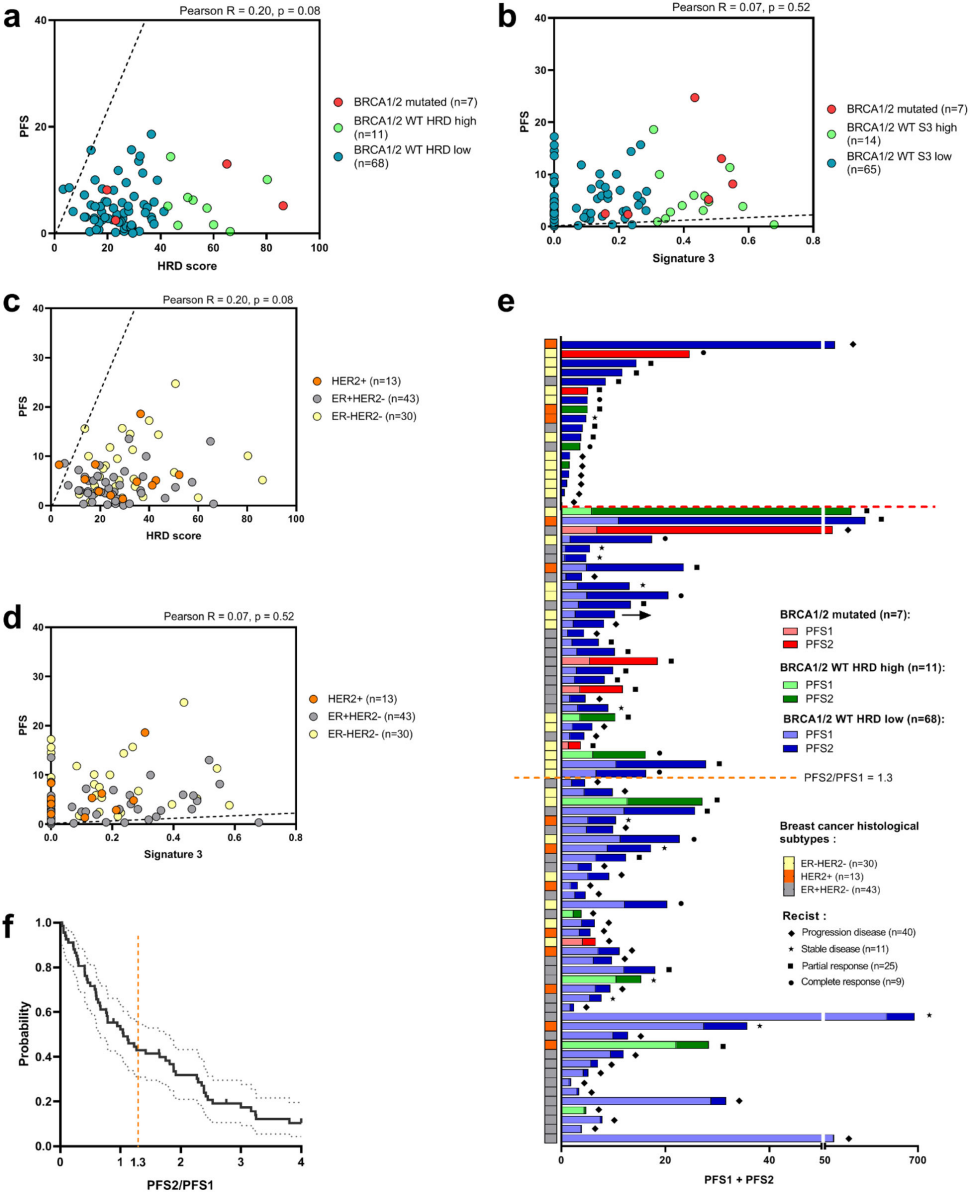
Association between HRD biomarkers and progression free survival. **a**, **b** Correlation between (**a**) HRD score or (**b**) S3 level and progression free survival (PFS), according to group formed with BRCA 1/2 mutated or HRD score/S3 level subgroups. Correlations were evaluated with Pearson’s correlation coefficient. **c**, **d** Correlation between (**c**) HRD score or (**d**) S3 level and progression free survival (PFS), according to breast cancer molecular subtypes. Correlations were evaluated with Pearson’s correlation coefficient. **e** Cumulative bar plots showing individual PFS1 and PFS2, ordered by descending PFS2/PFS1 ratio. The arrows denote censored data. Patients above the orange horizontal dashed line have a PFS2/PFS1 ratio >1.3. Patients above the red horizontal dashed line received platinum-based chemotherapy in first line. Bar colors represent BRCA 1/2 mutational and HRD status. Light colors were used for PFS1 (before platinum therapy) and dark colors for PFS2 (under platinum therapy). On the left, symbols represent platinum therapy response status and colors represent breast cancer molecular subtype. **f** Kaplan–Meier curve of PFS2/PFS1. Ticks denote censored data. Orange dashed line denotes PFS2/PFS1 > 1.3.

At the last follow-up, 80 patients had progressed or died, and the remaining 6 patients did not progress under platinum-based chemotherapy and were censored. For the overall cohort, median PFS obtained with platinum-based chemotherapy was 5.1 months, and median OS was 13.3 months. In our populations of interest, median PFS and OS were, respectively, 10.6 and 27.4 months in patients with *BRCA 1/2* mutated tumors, 6.3 and 14.9 months in patients with *WT* HRD-high tumors, and 4.2 and 12.3 months in patients with *WT* HRD-low tumors (Fig. 3A, B). Median PFS and OS were, respectively, 10.6 and 27.4 months in patients with *BRCA 1/2* mutated tumors, 4.4 and 16.8 months in patients with *WT* S3-high tumors, and 4.9 and 12.3 months in patients with *WT* S3-low tumors (Fig. 3C, D). We did not observe any significant correlation between HRD or S3 level, and PFS, in our cohort (Fig. 4a–d). These results are shown by the HRD biomarker subgroup in Fig. 4a, b, and by breast cancer molecular subgroup in Fig. 4c, d.

Again, no difference in terms of survival (PFS or OS) was observed in the population of patients who received first- or second-line platinum-based chemotherapy (whatever the cutoff used: 42, 33 or median value of our cohort) (Supplementary Fig. 7a–f).

Interestingly, in exploratory analyses, using median HRD score/S3 of our patient cohort as cutoff values, we observed better 1-year PFS in patients with BRCA mutated tumors, and a similar (albeit non statistically significant) trend for BRCA-WT HRD/S3-high tumors, when compared to BRCA-WT HRD/S3-low tumors (Supplementary Fig. 8a, b).

We next evaluated part of the clinical benefit as measured by the percentage of patients having PFS on platinum-based chemotherapy (PFS2) 1.3-fold longer than the PFS on prior systemic therapy for metastatic disease (PFS1). Nineteen patients received platinum-based chemotherapy in first line, and were thus excluded from this analysis (their PFS is nonetheless presented at the top of Fig. 4e).

Median PFS1 was 4.4 months, and median PFS2 was 5.1 months. The PFS2/PFS1 ratio was >1.3 in 43% of patients (29/68). A Kaplan–Meier plot of the PFS2/PFS1 ratio is illustrated in Fig. 4f. Individual PFS1 and PFS2 swimmer plots of the patients in all three groups (*BRCA* mutated, *WT* HRD-high and *WT* HRD-low) are presented in Fig. 4e. The results by S3 subgroup are reported in Supplementary Fig. 9. Importantly, the proportion of patients with a PFS2/PFS1 ratio >1.3 was higher in patients with *BRCA1/2* mutated tumors (80%) than in those with *BRCA WT* HRD-high tumors (37.5%), or *BRCA WT* HRD-low tumors (40%). However (probably due to the low number of BRCA-mutated cases), these proportions were not statistically significantly different between *BRCA* mutated, *WT* HRD-high and *WT* HRD-low tumors (mutated vs *WT* HRD-high: *p* = 0.27; mutated vs *WT* HRD-low: *p* = 0.16, Fisher’s exact test). The absence of difference was more marked between *WT* HRD-high and *WT* HRD-low tumors (*p* = 1 Fisher’s exact test). Similarly, no significant differences were observed between *WT* S3-high (46% of patients with PFS2/PFS1 ratio > 1.3), and *WT* S3-low tumors (38%, *p* = 0.75 Fisher’s exact test).

### PFS Cox models and clinical-biological factors associated with survival in patients treated with platinum-based chemotherapy

Using Cox models, we sought to identify the clinical or biological variables associated with PFS in our cohort of MBC patients treated with platinum-based chemotherapy.

By univariate analysis, we found that presence of liver metastases (HR: 2.43 [1.46; 4.05], *p* = 0.0006), and line of treatment in which platinum-based chemotherapy was received (HR: 1.13 [1.04; 1.23], *p* = 0.003), were significantly associated with shorter PFS. Conversely, histological subtype of breast cancer (ER−HER2− vs ER+HER2−: HR: 0.52 [0.32; 0.87] *p* = 0.01, and HER2+ vs ER+HER2−: HR: 0.51 [0.27; 0.99], *p* = 0.05), and HRD score (both as a continuous value or dichotomized according to the median value of the cohort; HR: 0.98 [0.97; 1], and HR: 0.62 [0.41; 1.01], respectively, *p* = 0.05) were associated with better PFS. Of note, neither HRD score dichotomized according to a cutoff of 42, nor S3 signature were significantly associated with PFS (Table 2).

**Table 2:** Factors associated with Progression-Free Survival in univariate and multivariate Cox analyses.

	Univariate			Multivariate		
Variable	HR	95% CI	*p*-value	HR	95% CI	*p*-value
HRD score (continuous)	0.98	[0.97; 1]	0.05	0.99	[0.97; 1]	0.18
HRD score (median, >26.25 vs ≤26.25)	0.62	[0.41; 1.01]	0.05			
HRD score >33 vs ≤33	0.67	[0.41; 1.08]	0.10			
HRD score >42 vs ≤42	0.62	[0.34; 1.13]	0.12			
Signature 3 (continuous)	0.38	[0.10; 1.51]	0.17			
Signature 3 (median, >0.14 vs ≤0.14)	0.74	[0.47; 1.15]	0.18			
Signature 3 (>0.30 vs ≤ 0.30)	0.72	[0.42; 1.24]	0.24			
Platinum line (continuous)	1.13	[1.04; 1.23]	0.003	1.08	[0.97; 1.21]	0.17
Number of metastatic sites (continuous)	1.13	[0.97; 1.33]	0.13			
BC molecular subtype (HER2+ vs ER+HER2−)	0.51	[0.27; 0.99]	0.05	0.51	[0.26; 1]	0.05
BC molecular subtype (ER−HER2− vs ER+HER2−)	0.52	[0.32; 0.87]	0.01	0.73	[0.41; 1.30]	0.28
WHO (0 vs ≥1)	0.92	[0.55; 1.56]	0.76			
Liver metastasis (yes vs no)	2.43	[1.46; 4.05]	0.0006	2.35	[1.36; 4.07]	0.002
*BRCA* WT vs mutated	1.97	[0.79. 4.91]	0.14	2.28	[0.81; 6.45]	0.12
**AUC**				0.92		

*HR* hazard ratio, *CI* confidence interval, *BC* breast cancer.

By multivariate analysis, in the model with the best predictive value (AUC: 0.92), the presence of liver metastases remained the only variable significantly associated with shorter PFS (HR: 2.35 [1.36; 4.07], *p* = 0.002) in our cohort (Table 2).

## Discussion

To the best of our knowledge, we report here the largest series reporting the efficacy of platinum-based chemotherapy in patients with various MBC subtypes, according to different genomic tests quantifying HRD, and all based on WES. Our results show that: (i) a small subset of *BRCA*-proficient MBC actually has genomic features associated with HR, at levels comparable to those of their *BRCA*-mutated counterparts; and (ii) these MBC patients with high HRD score or high S3 tumor level do not seem to benefit more from platinum-based chemotherapy than the others, in terms of response and/or PFS, regardless of BC molecular subtype.

Although MBC is not a curable disease, it is widely accepted that patients with metastatic disease should receive some form of chemotherapy with a view to reducing the burden of symptoms, while extending survival. Platinum-based chemotherapies are known to be effective for treating various cancer types, at the cost of sometimes significant toxicities (such as renal, neurologic, digestive, or hematological toxicities). Carboplatin and cisplatin have now become a routine part of the therapeutic arsenal commonly used for the treatment of MBC, and most patients receive this treatment during their medical history^14^. In MBC patients, platinum-based chemotherapy often combines carboplatin and gemcitabine, a regimen that offers a tolerable and effective treatment option, particularly for patients whose disease progressed after treatment with anthracyclines and/or taxanes^23–25^. Gemcitabine is an effective inhibitor of DNA repair. Synergistic cytotoxicyity may be induced by this association according to preclinical data, and gemcitabine may counteract platinum resistance caused by upregulation of DNA repair processes^25^. A meta-analysis^24^ of 6 clinical trials demonstrated the effectiveness of the association of cisplatin and gemcitabine in the first-line setting, but also in heavily pretreated patients, as in our clinical cohort.

However, a recent systematic review of randomized trials comparing platinum‐containing chemotherapy regimens with regimens not containing platinum in women with MBC, found that chemotherapy with platinum did not yield a major benefit, compared to platinum-free regimens, except perhaps for mTNBC patients^14^.

These results illustrate the need to refer to biomarkers associated with the benefit of platinum-based chemotherapy, in the context of the recent discovery of various tumor types harboring HRD, attribuable or not to *BRCA1/2* mutations. Around 15% of TNBC are characterized by HRD linked to germline or somatic *BRCA1/2* mutations^7^, and 40% are found to harbor HRD without *gBRCA1/2* mutation^2^, and could have a specific sensitivity to DNA-damaging agents. Thus, in a neoadjuvant setting, high pCR rates have been reported in patients treated by platinum monotherapy for *BRCA1/2* mutated or HR-deficient BC^8,9,19^. In localized TNBC, the impact of the addition of carboplatin/cisplatin into standard neoadjuvant chemotherapy (NAC) has been widely explored, and seems to increase clinical response and pCR rates^5,6,26^.

Different biological approaches have been developed to identify point mutations in homologous recombination repair (HRR) genes, or large genomic aberrations (“genomic scars”) and mutational signatures (like S3) associated with the presence of HRD, in order to select tumors that could benefit from DNA-damaging agents, such as PARP inhibitors or platinum salts^3,18,22,27^. In TNBC, pioneering studies suggested that the combined HRD score that we used in this study can predict response to platinum-containing NAC, even for *BRCA* proficient tumors^19^.

However, these results were obtained in prospective trials of NAC in TNBC patients, such as GeparSixto^6^, or BrighTNess^28^, in which tumors with high HRD scores had greater sensitivity to chemotherapy, but regardless of whether or not it contained platinum. In addition, for *BRCA* mutated patients, a meta-analysis^29^ (GeparSixto^26^, Inform^8^, and Brightness trials^30^) suggested that the addition of platinum to neoadjuvant chemotherapy did not significantly improve pathological complete response. One potential explanation was the superior response to anthracycline and alkylating agents, explaining the lack of additional benefit with platinum for *BRCA* mutation carriers. Finally, the recent publication of the TBCRC 030 trial^31^ reported that high-HRD scores (as in our study, with a cutoff value of 42 or 33) were not associated with pCR in patients receiving neoajuvant cisplatin monotherapy for early stage TNBC.

By contrast, fewer data are currently available for patients treated for metastatic disease, especially patients with non-TNBC subtypes. In mTNBC treated with platinum monotherapy (80% first line), the TBCRC 009 phase II trial^32^ reported higher HRD scores in responding patients, whatever the *BRCA 1/2* mutational status. However, these results were not confirmed in the TNT phase III trial^10^, which randomized mTNBC patients to first-line treatment with either docetaxel or carboplatin: higher response rates were observed with carboplatin in *gBRCA 1/2* mutated patients, but not in patients whose tumor harbored other HRD features, like high HRD score. To the best of our knowledge, there are currently no studies that have investigated the benefit of platinum-based chemotherapy in the metastatic setting beyond the first line, based on the results of genomic tests quantifying HRD using WES. This is very important in view of other studies that have tested more complex and expensive methods, such as whole genome sequencing (WGS). Indeed, HRDetect is a mutational-signature-based algorithm designed to detect “BRCAness” or HRD^33^. This tool was initially designed to accurately classify BC in their BRCA1/2 status, but can also identify HR-deficient tumors in BRCA-proficient BC^34^. Using HRDetect, Zhao et al. found that elevated HRDetect status was significantly associated with radiographic evidence of clinical improvement, but also with better survival and treatment duration in a small series of 33 mBC patients treated with platinum-based chemotherapies^35^. As in our study, they also found a small subset of patients with high HRDetect score, but without BRCA1/2 mutations. However, their results are in contrast with our conclusions, and these discrepancies could be explained by several factors. Firstly, we used target exome sequencing and WES data, which are likely not able to fully detect HRD status in some patients^34^. Secondly, the application of WGS in clinical practice is a very controversial topic, taking into consideration the financial costs, expertise and many technical issues. However, the continuing decrease in the cost of sequencing could enable more widespread use of WGS in years to come. This could make it possible to benefit from the other advantages of this technique, such as the integration of other markers of genomic instability, or of mutagenesis captured by structural variants^34^.

In our study, we did not apply HRDetect to our patients’ data, because the initial publication showed that the accuracy of this algorithm in detecting HRD from WES data is low compared to WGS^33^.

Moreover, commercially available signatures like the MyChoice HRD assay, assess LOH using informative SNPs outside the exome, and can be more informative than WES to determine LOH status^36^. For this reason, we decided to test several cutoff values in our study, including that given by the MyChoice signature, but also, for exploratory purposes, the median value of HRD score and S3 obtained in our cohort. These additional analyses tend to show a greater benefit of platinum-based chemotherapy when a lower HRD score cutoff (i.e. the median of our cohort) is used, which raises the question of the best cutoff value to consider in mBC, when WES is used. Similarly, in our exploratory analyzes, we show that signature 3 evaluated by SigMA is associated with better ORR, DCR and PFS, in contrast to what is observed using DeconstructSigs and COSMIC signatures identified by Alexandrov et al. In the original publication^37^, SigMA appears to capture HRD signatures with fewer numbers of mutations, possibly explaining greater robustness on WES data. Altogether, these results highlight the need for methodological optimization to properly ascertain HRD phenotype, as there are certain methods, when optimized, that seem to associate with platinum response, even in this small dataset.

One strength of our cohort, and a major difference with existing literature, is the inclusion of MBC other than solely the triple-negative subtype. Our results are in accordance with recent publications conducted in large cohort of BC patients with WGS approaches, and showing that HRDetect high scores were also observed in ER+ tumors^34^. Moreover, recent large genomic characterization of MBC reported both increased somatic genomic alterations in genes involved in HR pathway, and more HRD features (like increased S3 mutational signature) in MBC, as compared to early breast cancers (EBC), especially in the ER+/HER2− subtype^38^. Accordingly, we also found in our study that luminal MBC had higher HRD scores and S3 levels than eBC cases in TCGA cohort. Moreover, we also show in our study that a small subset of MBC harbored high HRD scores (≥42) and a high S3 mutational signature, at levels comparable to those of *BRCA 1/2* mutated tumors. This raises the question of whether these different tumors may benefit from similar therapeutic approaches.

Our results concerning platinum benefit are in accordance with previous results obtained in early and metastatic TNBC. Indeed, despite a trend for better response or PFS in *BRCA 1/2* mutated MBC compared to the others, we did not observe a link between HRD status (or S3 mutational signature), and PFS obtained with platinum-based chemotherapy (especially without any difference between HRD/S3-low and HRD/S3-high *BRCA*-proficient groups of tumors). This was also observed when examining the PFS2/PFS1 ratio: because PFS usually decreases over lines of treatment during the natural course of metastatic cancer^39^, we chose to examine this endpoint in order to compare patients who received platinum-based chemotherapy at different lines. A PFS2/PFS1 ratio >1.3 was proposed to define a treatment benefit in such patients^39^, and this ratio was recently used as a primary endpoint in the prospective MOSCATO 01 trial^40^.

Moreover, the analyses we carried out on patients who received first- or second-line platinum-based chemotherapy do not seem to show different results, despite a trend towards better response and survival in BRCA-mutated patients. Although this constitutes a small part of our cohort, it was important to analyze this aspect, since current evidence supports improved efficacy of PARP inhibitors when used early in mTNBC patients harboring gBRCA mutations^41^.

We believe our results are robust, and currently represent the largest series of patients reported to date, including MBC patients treated with platinum beyond the first line, and with various molecular subtypes. Furthermore, in our study, the vast majority of tumor samples analyzed (78%) stemmed from biopsies of metastases, and not from the primary tumor, which probably better represents the molecular status of the metastatic disease.

Conversely, our study also has some limitations such as the single-center nature of the study, the small number of patients analyzed, and the heterogeneity of diseases. Additionally, low coverage of certain samples in our cohort may limit mutational signature inference. However, these conditions reflect the real-life analyses that are done in a cancer center on the population of breast cancer patients recruited. Due to the small number of patients with BRCA mutation in our study, we cannot exclude the possibility that the lack of benefit from platinum observed in this population is due to a lack of statistical power.

It is also important to note that one of the major limitations of the biological tests we used, assessing genomic scars, is that they cannot detect restoration of functional HR (which is a resistance mechanism that can appear under therapeutic pressure)^18^. To limit this bias as much as possible, we excluded from our series all patients previously treated with platinum and/or PARP inhibitors. However, given the very advanced stage of the MBCs studied, it cannot be ruled out that such a phenomenon could have biased our results. It would be useful to incorporate functional biomarkers, such as evaluation of *RAD51* foci^42^, as a predictive biomarker of functional HR. A further limitation of our work is the relatively small number of patients included (especially *BRCA* mutated tumors as control group), and the single-center recruitment, which may impact the representativity of patients and the statistical significance, especially for analyses conducted in different BC subtypes. Thus, our results should be considered as descriptive and exploratory, and warrant confirmation in further studies with larger sample sizes. Nevertheless, this cohort included all consecutive MBC patients treated with platinum-based chemotherapy since the implementation of NGS in our center.

Finally, the only factor independently associated with survival in our cohort was the presence of hepatic metastases. This result is not very surprising because it is a well-described prognostic factor in MBC^43^. As polychemotherapies (for example with carboplatin-gemcitabine, as in our cohort) are more frequently used for patients presenting a visceral crisis, we retrospectively analyzed the clinical records of the patients, which made it possible to show that very few patients were in visceral crisis (*N* = 10; 12%) at the time of the prescription of the platinum-based chemotherapy, and that our series was therefore not biased by this type of recruitment.

In conclusion, based on standard and currently used methods/cutoff values, our study does not support the use of WES for quantifying HRD to preferentially decide on platinum-based chemotherapy treatment in heavily pretreated MBC, regardless of the breast cancer subtype. However, some of our exploratory analyses could show that tumor exome sequencing methodology has to be approached systematically, and further validated/ standardized prior to its clinical use. Additional translational research incorporating other genomic tools is necessary to reveal whether HRD status outside of BRCA mutations may be associated with platinum/PARP inhibitor response.

## Methods

### Study population and clinical endpoints

Eighty-six patients with MBC in whom WES (Whole Exome Sequencing) analysis was performed and interpreted according to the Molecular Tumor Board of the Georges François Leclerc Cancer Center were included in this single-center, retrospective study. Patients were enrolled between June 2018 and March 2020, and all received at least one line of platinum-based chemotherapy. Platinum chemotherapy (carboplatin or cisplatin-based) was frequently associated with gemcitabine according to local practice.

Radiological response to platinum-based chemotherapy was determined locally, and assessed by clinical and radiological evaluations every 2-4 months for each patient. The Response Evaluation Criteria in Solid Tumors, version 1.1 (RECIST 1.1), were used to assess treatment efficacy for measurable or evaluable lesions. Complete response (CR) was defined as the disappearance of all lesions. Partial response (PR) was defined as a decrease ≥30% over baseline in the sum of diameters of target lesions, and for non-measurable skin lesions by a clinical decrease in the size of lesions. Progression (PD: progressive disease) was defined as an increase ≥20% in the smallest sum of diameters as reference, or new clinical or radiological lesions. Stable disease (SD) was defined as no signs of progression or response, after 3 months of treatment. We also assessed disease control rate (DCR), which includes the percentage of patients with CR, PR and SD, and the objective response rate (ORR), which includes the percentage of patients with CR or PR.

WES analysis is performed as part of routine care in our center in order to find potential targetable mutations for second-line therapy. Before patients consented to WES of their tumoral tissue, they were informed by their oncologist. Only patients from whom informed consent was obtained and recorded in the medical chart were included in this retrospective study. The study was approved by the CNIL (French national commission for data privacy) and the local ethics committee, and was performed in accordance with the Helsinki Declaration and European legislation.

### Sample selection

Pathologists selected an archival formalin-fixed paraffin-embedded (FFPE) tumor sample (primary or metastasis) for genomic analyses. Only 19 (22%) samples came from primary tumors, and 67 (78%) from a metastatic site biopsy. Tumor cellularity was assessed by a senior pathologist on a hematoxylin and eosin slide from the same biopsy core used for nucleic acid extraction and molecular analyses. Except for patients reported as germline-BRCA mutated, 40 patients were tested on tumor alone, and 46 patients also had blood tested for germline pathogenic mutation.

### TCGA patient material

RNAseqV2 data with RSEM normalization, MAF and corresponding clinical data were downloaded from the TCGA data portal (https://portal.gdc.cancer.gov/). HRD scores and the 3 components of HRD/genome scarring scores, namely HRD-Loss of heterozygosity (LOH), Large Scale Transition (LST) and Telomeric Allelic Imbalance (TAI) were calculated as per Knijnenburg et al.^44^.

### DNA isolation

DNA was isolated from archival tumor tissue using the Maxwell 16 FFPE Plus LEV DNA purification kit (Promega, Madison, WI, USA). DNA from whole blood (germline DNA) was isolated using the Maxwell 16 Blood DNA Purification kit (Promega) following the manufacturer’s instructions. The quantity of extracted genomic DNA was assessed by a fluorometric method with a Qubit device.

### Whole-exome capture and sequencing

Two hundred ng of genomic DNA were used for library preparation, using the Agilent SureSelectXT reagent kit (Agilent Technologies, Santa Clara, CA, USA). The totality of the enriched library was used in the hybridization and captured with the SureSelect All Exon v5 or v6 (Agilent Technologies) baits. Following hybridization, the captured libraries were purified according to the manufacturer’s recommendations and amplified by polymerase chain reaction (12 cycles). Normalized libraries were pooled, and DNA was sequenced on an Illumina NextSeq500 device using 2 ×111-bp paired-end reads and multiplexed. More than 90% of the target sequence was covered with a read depth of at least 10X for somatic DNA (Supplementary Table 3).

### Exome analysis pipeline

Reads in FASTQ format were aligned to the reference human genome GRCh37 using the Burrows–Wheeler aligner (BWA v.0.7.15). Local realignment was performed using the Genome Analysis Toolkit (GATK v.3.6). Duplicate reads were removed using Picard v.2.5. To identify somatic single-nucleotide variants (SNVs), a validated pipeline was used that integrates mutation calls from three different mutation callers. Single-Nucleotide Variants (SNVs) were called with VarScan (v2.4.3)^45^ and Mutect (v1.1.7)^46^, and insertion/deletions (indels) were called with VarScan and Strelka (v2.9.2)^47^. SNV signatures were generated using DeconstructSigs (v1.8.0)^48^ and COSMIC signatures identified by Alexandrov et al.^49^. To confirm corresponding results, signature 3 was also evaluated using SignatureAnalyser (v1.1)^50,51^, and SigMa^37^. In this study, we evaluated the combined homologous recombination deficiency score (HRD score) defined as the unweighted numeric sum of LOH (loss of heterozygosity)^22^, TAI (telomeric allelic imbalance)^20^, and LST (large-scale state transitions)^21^. HRD score was obtained through the scarHRD pipeline^36^.

Variant pathogenicity was evaluated according to the current guidelines for reporting variants in cancer^52^. In particular, variants located in tumor suppressor genes were evaluated in accordance with the ACMG guidelines and classified into 5 classes of pathogenicity^53^. The ACMG frame used includes 26 criteria and the associated decision algorithm leading to the actual 5 classes of variants: Class 1 (benign) to Class 5 (pathogenic). The databases used to document the population frequency are non-cancer GnomAD v 3.1 maximal subpopulation frequencies with a frequency cutoff of 0.001.

The databases used to screen published pathogenic mutations are Clinvar (online version, SCV000020145 version, addressing only 3 stars classified variants), Swissprot, LOVD and UMD, using the data concerning class 4 and 5 variants solely as a ACMG PP5 criterion. We add the use of the French COVAR Consortium database using the data concerning class 4 and 5 variants as a ACMG PS1 criterion. Finally, we use our local database using the data concerning class 4 and 5 with conservation of the same class. The databases used for pathogenicity predictions are AlignGVGD, SIFT, Polyphen, and Mutation Taster, concerning missense variants. We use Splice-Site finder-like, MAxEntScan, NNSPLICE, and GeneSplicer concerning splice-site variants.

### Statistical analysis

Patient and disease characteristics were compared between the different groups of interest using the Chi square or Fisher’s exact test for qualitative variables and the Wilcoxon test for continuous variables, as appropriate. BRCA1/2 mutation status, and HRD and signature 3, respectively, were used to stratify patients into three groups. Patients with a *BRCA1/2* mutation were considered in the “mutated group”. When using HRD, patients wild type for *BRCA1/2* and with a low HRD (<42) were classed in the “*WT* HRD-low” group, and patients wild type for *BRCA1/2* with a high HRD (≥42) were in the “*WT* HRD-high” group. The cutoff value of 42 was chosen according to the classical readout of this assay (“myChoice HRD”), as published elsewere^18^. An exploratory cutoff value of 33, recently evaluated in the BrighTNess trial^28^, and the TBCRC 030 study^31^ was also tested. As HRD is only approximated by WES, we also used the median value of HRD scores obtained in our cohort as an additional exploratory cutoff value. The same method was used to stratify the patients with signature 3, using a cutoff value of 0.30^54^, or the median value obtained in our cohort; the groups were, respectively, “*BRCA* mutated”, “*WT* S3-low” and “*WT* S3-high”.

The same methods were used to stratify the patients with signature 3 when using deconstructSigs, using a cutoff value of 0.30^54^ or the median value obtained in our cohort. For SignatureAnalyzer, only the median value was used; the groups were, respectively, “*BRCA* mutated”, “*WT* S3-low” and “*WT* S3-high”. With SigMa, patients are directly classified as Signature 3-positive or -negative by the tool.

Survival analysis was performed using the survival R library. Continuous variables were dichotomized using the methodology of Lausen et al through the maxstat library^55^. The prognostic value of the different variables was tested using univariate Cox regression for PFS1. Significant variables and the BRCA mutational status were selected to fit a multivariate model. PFS1 was defined by the European Medicines Agency (EMA) as the time since the start of the last treatment prior to progression, as defined by RECIST 1.1 or clinical progression. PFS2 was defined as the time from the start of platinum therapy to progression, as defined by RECIST 1.1, clinical progression, or death from any cause. PFS2 was compared with PFS1 for each patient using the PFS2/PFS1 ratio (or growth modulation index^56^). Overall survival (OS) was defined as the time from the start of platinum therapy to death from any cause. Patients alive or lost to follow-up were censored at the date of the last follow-up. Survival probabilities were estimated using the Kaplan–Meier method and survival curves were compared using the log-rank test. Statistical analyses were performed using R software ((http://www.R-project.org/) and graphs were drawn using GraphPad Prism version 7.03 (GraphPad Software, LLC, San Diego, USA).

### Reporting summary

Further information on research design is available in the Nature Research Reporting Summary linked to this article.

## Supplementary information

Supplemental material

Reporting Summary

## Data Availability

Raw data and associated clinical data used in this paper are available on SRA repository under BioProject accession number PRJNA793752.

## Code availability

Code developed for the analysis is available upon request.
