# Supplementary material for: Efficacy of platinum-based chemotherapy in metastatic breast cancer and HRD biomarkers: utility of exome sequencing
Source: NPJ Breast Cancer. 2022 Mar 4;8:28. doi: 10.1038/s41523-022-00395-0 (PMC8897409; doi:10.1038/s41523-022-00395-0)
Supplement: Supplementary file 1 — Supplemental material [file 41523_2022_395_MOESM1_ESM.pdf]

## Supplementary figures

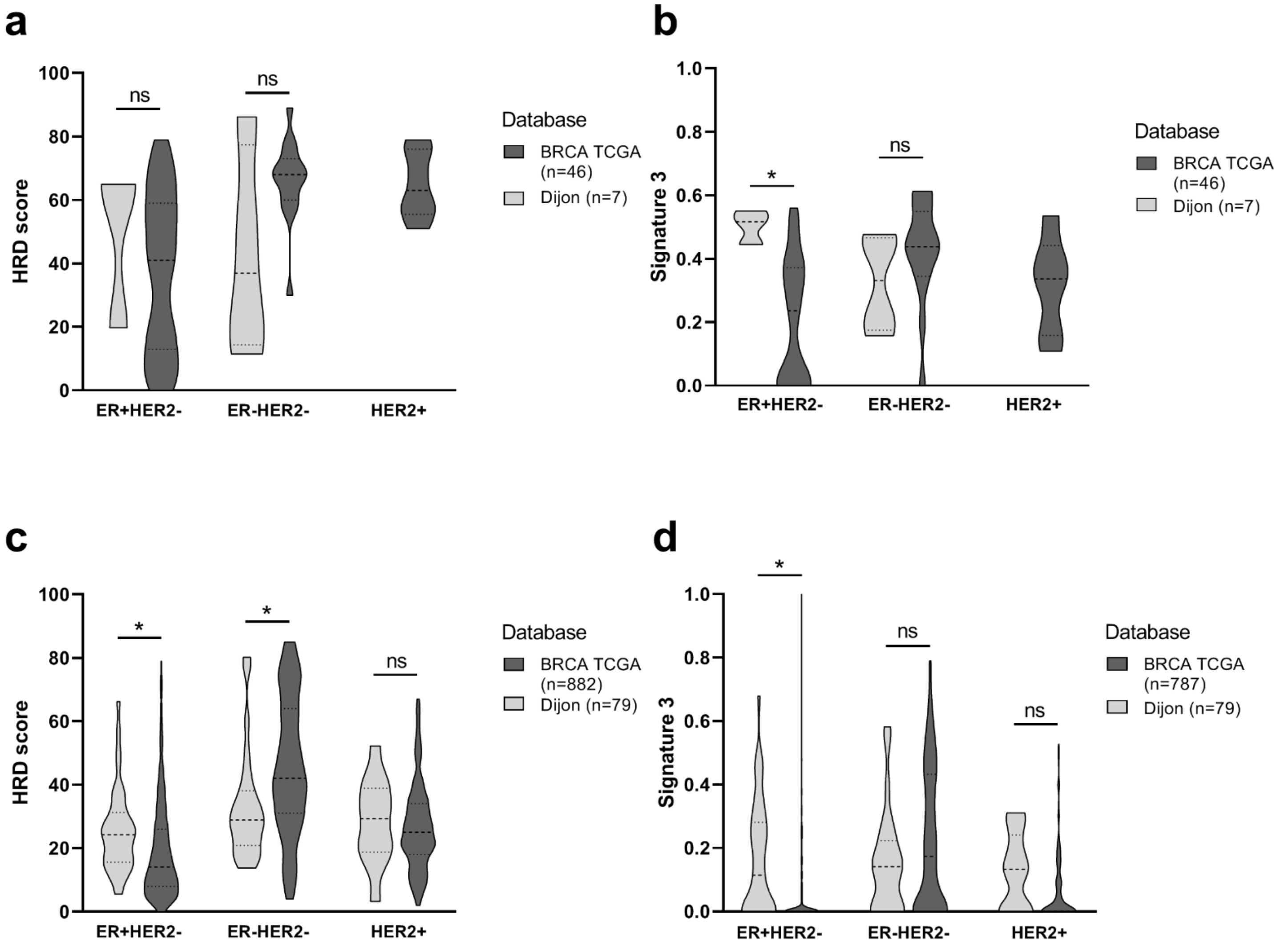


**Supplementary Figure 1:**

**a-b.** Violin plots representing in *BRCA* mutated tumors, the distribution of HRD score (a) and signature 3 level (b) according to breast cancer molecular subtype and cohort, namely patients from our cohort treated with platinum-based chemotherapy (Dijon) and *BRCA* TCGA cohorts.

**c-d:** Violin plots representing in *BRCA* WT tumors, the distribution of HRD score (c) and signature 3 level (d) according to breast cancer molecular subtype and cohort, namely patients from our cohort treated with platinum-based chemotherapy (Dijon) and *BRCA* TCGA cohorts. * : Significant Wilcoxon test p-value.

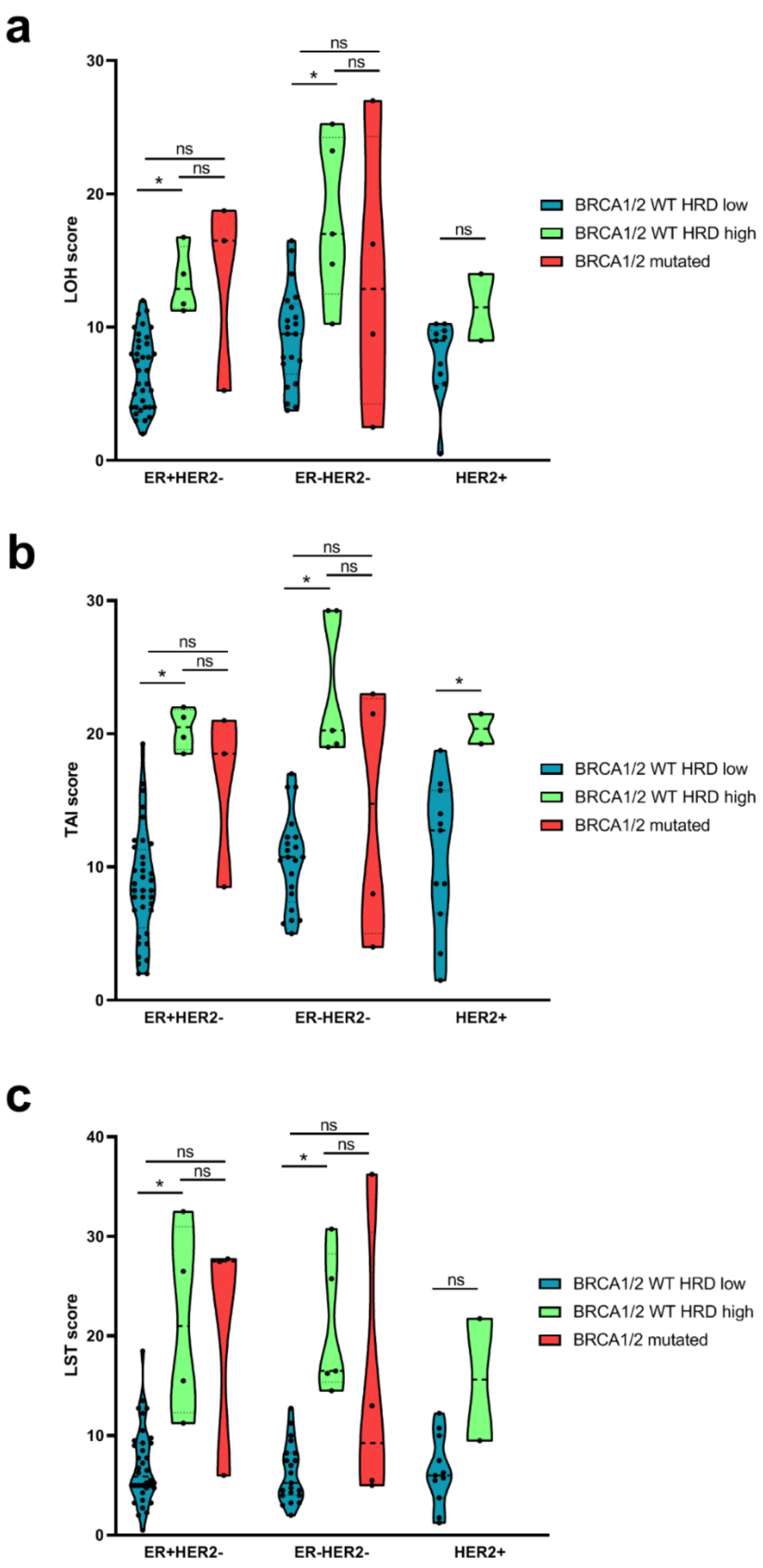


**<u>Supplementary Figure 2:</u>**

**a-c.** Violin plots representing the distribution of LOH (a), TAI (b) and LST (c) scores according to breast cancer molecular subtype and *BRCA 1/2* mutational status, or HRD score status. Patients were stratified in three groups for each molecular subtype: *BRCA 1/2* mutated patients (red), *BRCA 1/2* wild-type with high HRD score (green) and *BRCA 1/2* wild-type with low HRD score (blue). * : p value of Wilcoxon test was significant.

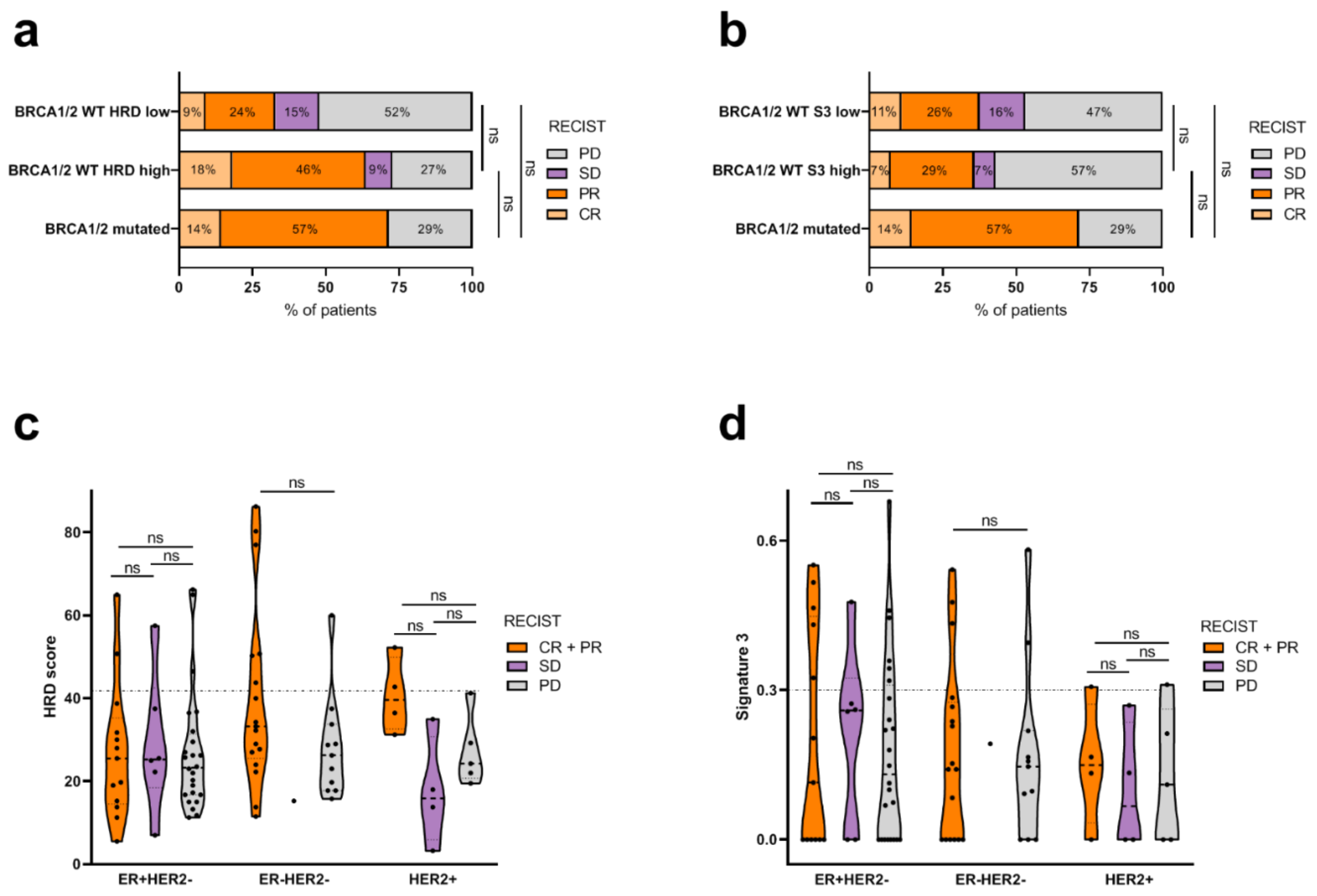


**Supplementary Figure 3:**

**a-b.** Cumulative bar plots separately showing the proportion of patients with complete response (CR), partial response (PR), stable disease (SD) or progressive disease (PD), represented in each group for *BRCA 1/2* mutated and (a) HRD status or (b) signature 3 level. * : significant Fisher's exact test p-value.

**c-d.** Violin plots representing the distribution of HRD score (c) and signature 3 level (d) according to breast cancer molecular subtype and response to platinum chemotherapy.

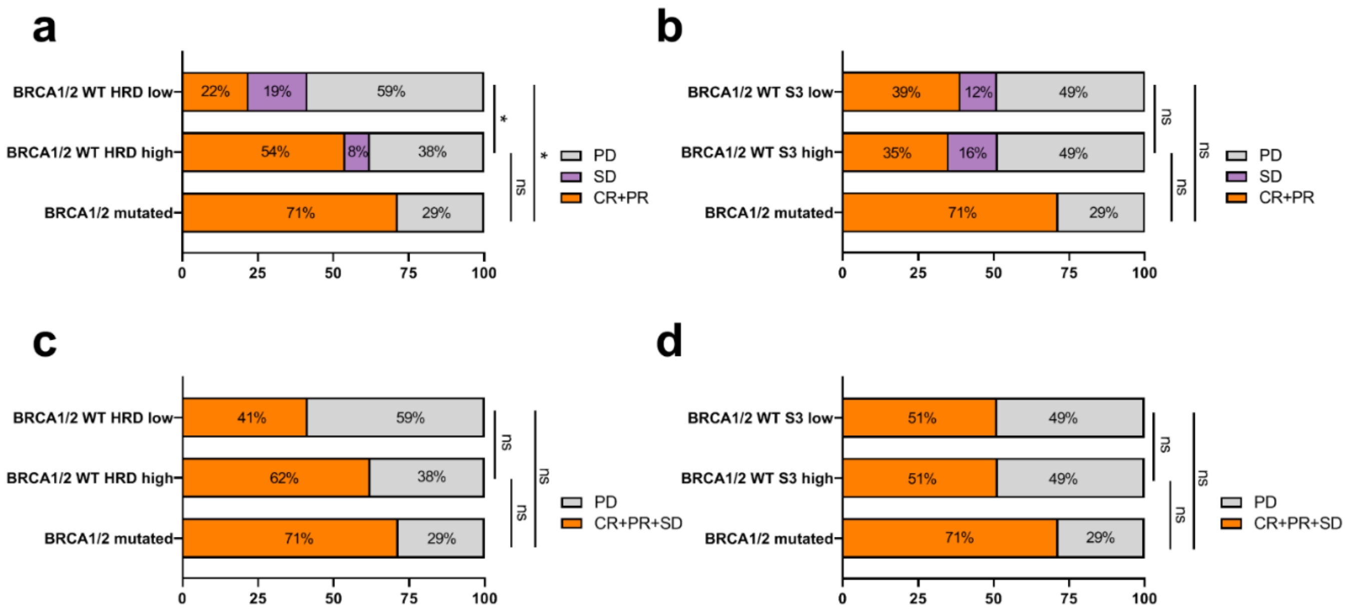


**Supplementary Figure 4:**

**a-b.** Cumulative bar plots showing ORR using HRD and signature 3 median values as cutoff : proportions of patients with complete response (CR) + partial response (PR), or stable disease (SD) or progressive disease (PD) are represented in each group formed with *BRCA 1/2* mutated and (a) HRD status, or (b) signature 3 level. * : significant Fisher's exact test p-value.

**c-d.** Cumulative bar plots showing DCR using HRD and signature 3 median values as cutoff : proportions of patients with complete response (CR) + partial response (PR) + stable disease (SD), or progressive disease (PD) are represented in each group formed with *BRCA 1/2* mutated and (c) HRD status or (d) signature 3 level * : significant Fisher's exact test p-value

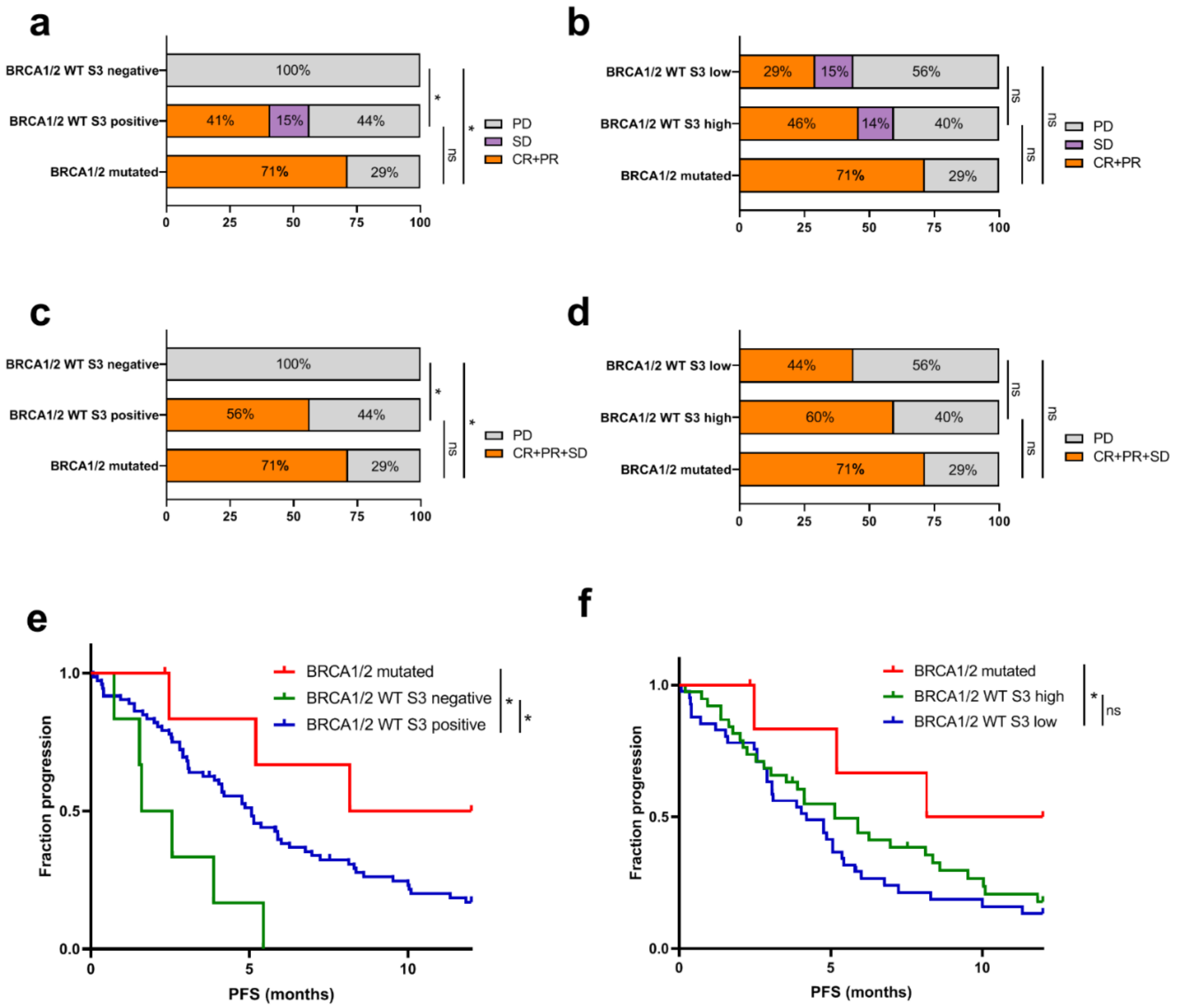


**Supplementary Figure 5:**

**a-b.** Cumulative bar plots showing ORR using signature 3 performed with Sigma (a) and Signature Analyzer (b): proportions of patients with complete response (CR) + partial response (PR), or stable disease (SD) or progressive disease (PD) are represented in each group formed with *BRCA 1/2* mutated and signature 3 level. * : significant Fisher's exact test p-value.

**c-d.** Cumulative bar plots showing DCR using signature 3 performed with Sigma (c) and Signature Analyzer (d): proportions of patients with complete response (CR) + partial response (PR) + stable disease (SD), or progressive disease (PD) are represented in each group formed with *BRCA 1/2* mutated and signature 3 level. * : significant Fisher's exact test p-value

**e-f.** Kaplan–Meier curves of progression free survival according to *BRCA 1/2* mutated and signature 3 level performed with Sigma (e) and Signature Analyzer (f). Red curves: patients with *BRCA* mutated tumors, green curves: patients with *BRCA WT* S3-high tumors, blue curves: patients with *BRCA WT* S3-low tumors.

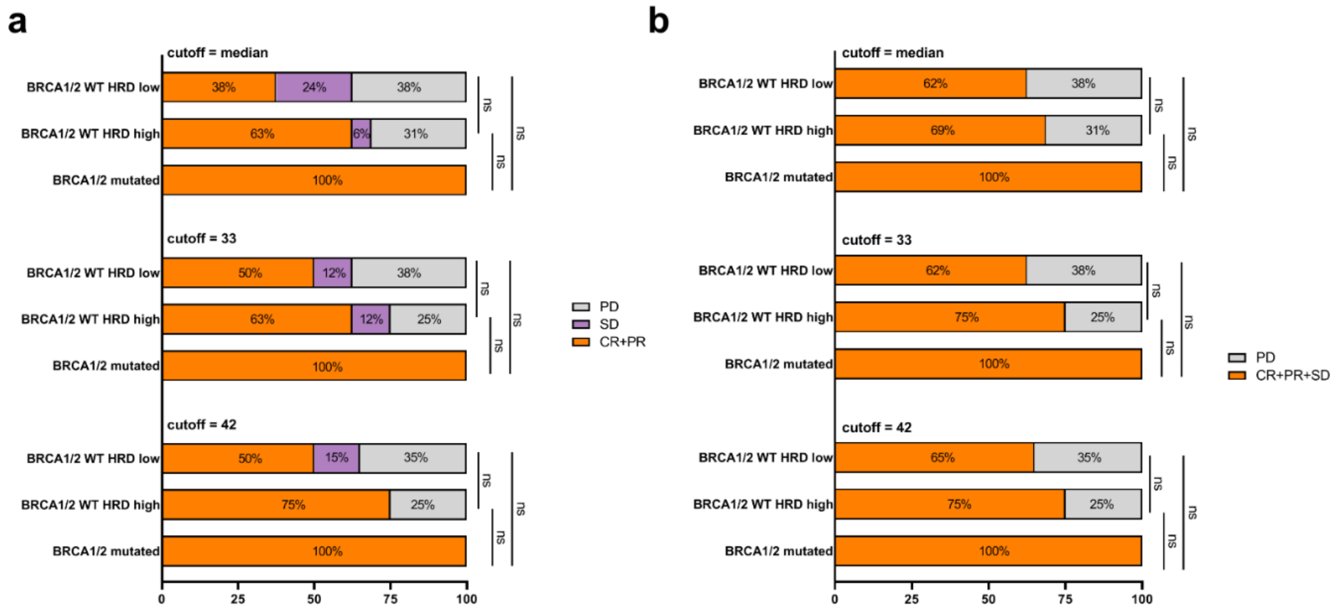


**Supplementary Figure 6:**

**a.** Cumulative bar plots showing ORR using 42, 33 and median values as cutoff for HRD score: proportions of patients who received first- or second-line platinum-based chemotherapy with complete response (CR) + partial response (PR), or stable disease (SD) or progressive disease (PD) are represented in each group formed with *BRCA 1/2* mutated and HRD status. ns : non significant Fisher's exact test p-value.

**b.** Cumulative bar plots showing DCR using 42, 33 and median values as cutoff for HRD score : proportions of patients who received first- or second-line platinum-based chemotherapy with complete response (CR) + partial response (PR) + stable disease (SD) or progressive disease (PD) are represented in each group formed with *BRCA 1/2* mutated and HRD status. ns : non significant Fisher's exact test p-value.

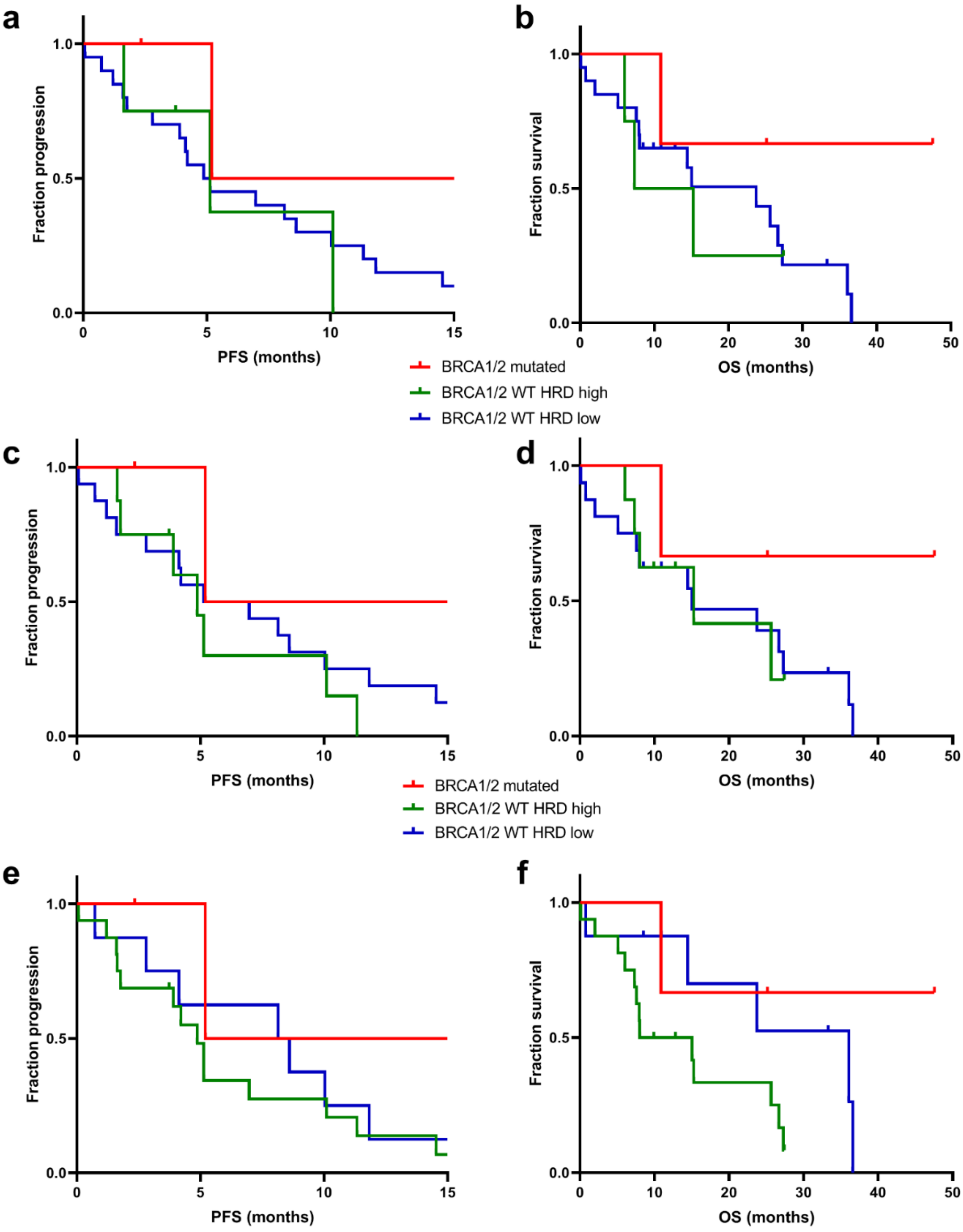


## Supplementary Figure 7:

**a-b.** Kaplan–Meier curves of progression free survival (a) and overall survival (b) for patients who received first- or second-line platinum-based chemotherapy, according to *BRCA 1/2* mutated and HRD score level. Red curves: patients with *BRCA* mutated tumors, green curves: patients with *BRCA WT* HRD-high tumors, blue curves: patients with *BRCA WT* HRD-low tumors. The HRD cutoff used was 42. Ticks denote censored data.

**c-d.** Kaplan–Meier curves of progression free survival (c) and overall survival (d) for patients who received first- or second-line platinum-based chemotherapy, according to *BRCA 1/2* mutated and HRD score level. Red curves: patients with *BRCA* mutated tumors, green curves: patients with *BRCA WT* HRD-high tumors, blue curves: patients with *BRCA WT* HRD-low tumors. The HRD cutoff used was 33. Ticks denote censored data.

**e-f.** Kaplan–Meier curves of progression free survival (e) and overall survival (f) for patients who received first- or second-line platinum-based chemotherapy, according to *BRCA 1/2* mutated and HRD score level. Red curves: patients with *BRCA* mutated tumors, green curves: patients with *BRCA WT* HRD-high tumors, blue curves: patients with *BRCA WT* HRD-low tumors. The HRD cutoff used was median value. Ticks denote censored data.

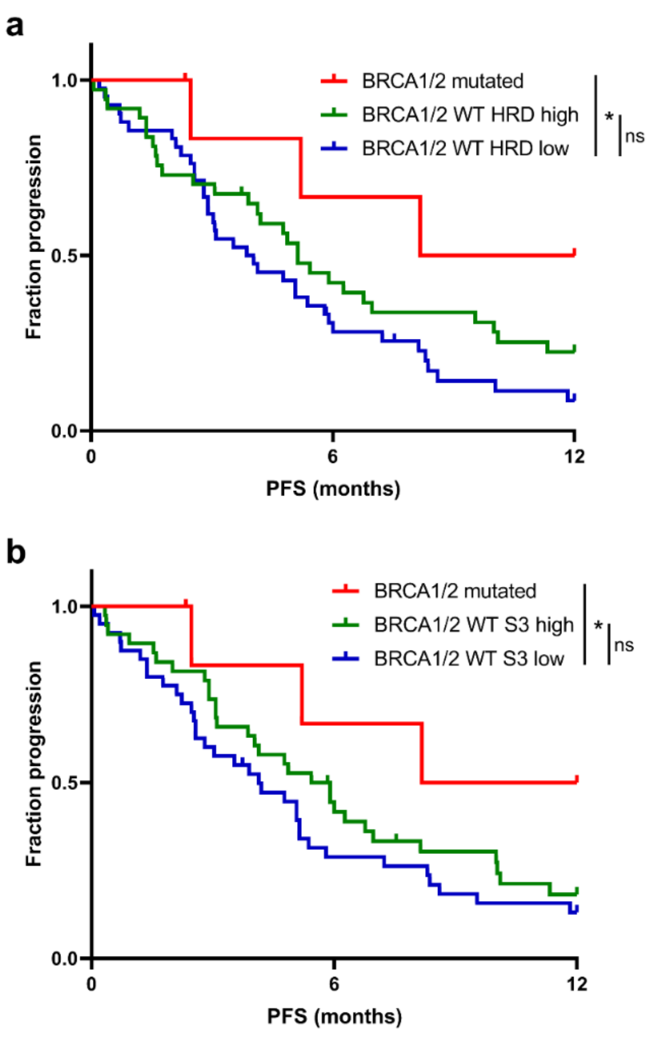


**<u>Supplementary Figure 8:</u>**

**a-b.** Kaplan–Meier curves of progression free survival for patients treated with platinum-based chemotherapy, according to *BRCA 1/2* mutated and HRD score level (a) or signature 3 level (b) . Red curves: patients with *BRCA* mutated tumors, green curves: patients with *BRCA WT* HRD / S3 -high tumors, blue curves: patients with *BRCA WT* HRD / S3 -low tumors. The cutoff used were median value for HRD score and signature 3. Ticks denote censored data.

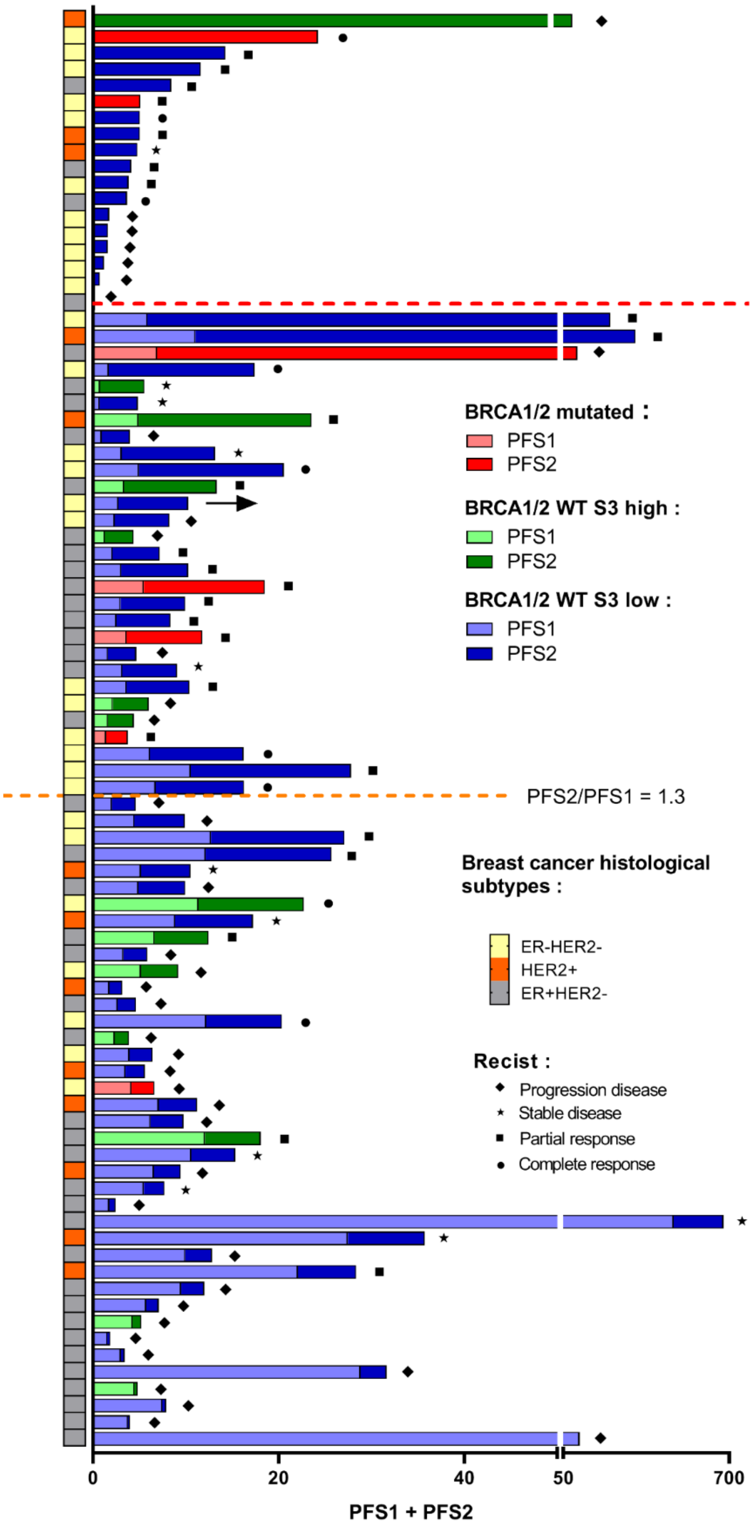


**Supplementary Figure 9:**

Cumulative bar plots showing individual PFS1 and PFS2, ordered by descending PFS2/PFS1 ratio. The arrow denotes censored data. Patients above the orange horizontal dashed line have a PFS2/PFS1 ratio > 1.3 and patients above the red horizontal dashed line received platinum-based chemotherapy in first line. Bar colors represent *BRCA 1/2* mutational and S3 level status. Light colors were used for PFS1 (before platinum therapy) and dark colors for PFS2 (under platinum therapy). On the left, symbols represent platinum therapy response status and colors represent breast cancer molecular subtype.

# Supplementary tables

**<u>Supplementary table 1:</u>** Detailed of pathogenic gene variations involved in homologous recombination

| | HRD score | S3 | BC subtype | Gene mutation | Mutation type | Biallelic Mutation |
|---|---|---|---|---|---|---|
| **N17-18** | 36.5 | 0.31 | HER2+ | ATM | germline | no |
| **N19-278** | 22 | 0.31 | HER2+ | ATM | germline | yes |
| **N16-160** | 19.75 | 0.15 | ER-HER2- | ATR | somatic | no |
| **N16-26** | 11.5 | 0.23 | ER-HER2- | BRCA1 | germline | no |
| **N16-71** | 23 | 0.16 | ER-HER2- | BRCA1 | somatic | yes |
| **N17-388** | 50.75 | 0.43 | ER-HER2- | BRCA1 | somatic | yes |
| **N18-215** | 86.25 | 0.48 | ER-HER2- | BRCA1 | germline | yes |
| **N18-168** | 65 | 0.52 | ER+HER2- | BRCA2 | germline | yes |
| **N19-212** | 65 | 0.45 | ER+HER2- | BRCA2 | germline | yes |
| **N19-213** | 19.75 | 0.55 | ER+HER2- | BRCA2 | germline | no |

**Supplementary table 2 :** Output metrics for SigMA analysis

| Sample | total_snvs | categ | Signature_3_ml | Signature_3_c | exp_sig3 | Signature_3_l_rat | Signature_3_mva | pass_mva | pass_mva_strict |
|---|---|---|---|---|---|---|---|---|---|
| N15-170 | 8775 | Signature_3_hc | 1 | 0.67 | 3529.81 | 1.00 | 0.99 | TRUE | TRUE |
| N15-300 | 5315 | Signature_3_hc | 1 | 0.67 | 1441.43 | 1.00 | 0.97 | TRUE | TRUE |
| N15-308 | 5312 | Signature_3_hc | 1 | 0.71 | 1370.23 | 1.00 | 0.96 | TRUE | TRUE |
| N15-314 | 5240 | Signature_3_hc | 1 | 0.69 | 1313.63 | 1.00 | 0.95 | TRUE | TRUE |
| N15-337 | 6662 | Signature_3_hc | 1 | 0.69 | 2664.15 | 1.00 | 0.99 | TRUE | TRUE |
| N15-375 | 10692 | Signature_3_hc | 1 | 0.64 | 6052.27 | 1.00 | 0.99 | TRUE | TRUE |
| N15-399 | 6898 | Signature_3_hc | 1 | 0.67 | 2443.21 | 1.00 | 0.99 | TRUE | TRUE |
| N15-451 | 7928 | Signature_3_hc | 1 | 0.62 | 3609.12 | 1.00 | 0.99 | TRUE | TRUE |
| N15-465 | 7072 | Signature_3_hc | 1 | 0.68 | 2624.79 | 1.00 | 0.99 | TRUE | TRUE |
| N15-481 | 7481 | Signature_3_hc | 1 | 0.68 | 2723.18 | 1.00 | 0.99 | TRUE | TRUE |
| N15-486 | 7231 | Signature_3_hc | 1 | 0.66 | 3104.80 | 1.00 | 0.99 | TRUE | TRUE |
| N15-506 | 6344 | Signature_3_hc | 1 | 0.67 | 1989.52 | 1.00 | 0.99 | TRUE | TRUE |
| N15-76 | 9057 | Signature_3_hc | 1 | 0.65 | 3802.03 | 1.00 | 0.99 | TRUE | TRUE |
| N16-117 | 9651 | Signature_3_hc | 1 | 0.70 | 5424.43 | 1.00 | 0.99 | TRUE | TRUE |
| N16-160 | 6281 | Signature_3_hc | $< 10^{-3}$ | 0.67 | 1664.41 | 1.00 | 0.53 | TRUE | TRUE |
| N16-192 | 7108 | Signature_3_hc | 1 | 0.69 | 3152.80 | 1.00 | 0.99 | TRUE | TRUE |
| N16-26 | 6942 | Signature_3_hc | 1 | 0.72 | 2818.53 | 1.00 | 0.99 | TRUE | TRUE |
| N16-260 | 6946 | Signature_3_hc | 1 | 0.65 | 2895.73 | 1.00 | 0.99 | TRUE | TRUE |
| N16-71 | 9829 | Signature_3_hc | 1 | 0.67 | 6269.46 | 1.00 | 0.99 | TRUE | TRUE |
| N17-05 | 5467 | Signature_clock: Signature_3_l_rat | $< 10^{-3}$ | 0.68 | 1117.06 | 1.00 | 0.08 | FALSE | FALSE |
| N17-149 | 8505 | Signature_3_hc | 1 | 0.76 | 2862.77 | 1.00 | 0.99 | TRUE | TRUE |
| N17-214 | 6936 | Signature_3_hc | 1 | 0.70 | 3266.11 | 1.00 | 0.99 | TRUE | TRUE |
| N17-251 | 7898 | Signature_3_hc | 1 | 0.74 | 3982.34 | 1.00 | 0.99 | TRUE | TRUE |
| N17-296 | 7411 | Signature_3_hc | 1 | 0.68 | 3303.67 | 1.00 | 0.99 | TRUE | TRUE |
| N17-398 | 7103 | Signature_3_hc | 1 | 0.73 | 2612.03 | 1.00 | 0.99 | TRUE | TRUE |
| N17-402 | 7491 | Signature_3_hc | 1 | 0.73 | 2856.97 | 1.00 | 0.99 | TRUE | TRUE |
| N17-412 | 8127 | Signature_3_hc | 1 | 0.66 | 4259.14 | 1.00 | 0.99 | TRUE | TRUE |
| N18-119 | 5595 | Signature_3_hc | 1 | 0.69 | 2386.08 | 1.00 | 0.99 | TRUE | TRUE |
| N18-139 | 7476 | Signature_3_hc | 1 | 0.71 | 2496.50 | 1.00 | 0.99 | TRUE | TRUE |
| N18-156 | 6753 | Signature_3_hc | 1 | 0.72 | 2185.12 | 1.00 | 0.99 | TRUE | TRUE |
| N18-174 | 8251 | Signature_3_hc | 1 | 0.72 | 3416.54 | 1.00 | 0.99 | TRUE | TRUE |
| N18-235 | 8750 | Signature_3_hc | 1 | 0.71 | 4684.04 | 1.00 | 0.99 | TRUE | TRUE |
| N18-242 | 7738 | Signature_3_hc | 1 | 0.68 | 3699.32 | 1.00 | 0.99 | TRUE | TRUE |
| N18-281 | 7365 | Signature_3_hc | 1 | 0.74 | 2545.79 | 1.00 | 0.99 | TRUE | TRUE |
| N18-308 | 8032 | Signature_3_hc | 1 | 0.71 | 3036.58 | 1.00 | 0.99 | TRUE | TRUE |
| N18-365 | 8112 | Signature_3_hc | 1 | 0.74 | 2977.94 | 1.00 | 0.99 | TRUE | TRUE |
| N18-373 | 10875 | Signature_3_hc | 1 | 0.74 | 5790.83 | 1.00 | 0.99 | TRUE | TRUE |
| N19-278 | 11132 | Signature_3_hc | 1 | 0.60 | 7490.95 | NA | 0.99 | TRUE | TRUE |
| N19-291 | 10316 | Signature_3_hc | 1 | 0.60 | 5813.80 | NA | 0.99 | TRUE | TRUE |
| N19-62 | 11083 | Signature_3_hc | 1 | 0.77 | 4794.06 | 1.00 | 0.99 | TRUE | TRUE |
| N15-346 | 391 | Signature_3_lc | 1 | 0.67 | 0.00 | 0.00 | 0.36 | TRUE | FALSE |
| N15-408 | 446 | Signature_3_lc | 0.47 | 0.64 | 3.16 | 0.52 | 0.46 | TRUE | FALSE |
| N15-478 | 310 | Signature_3_hc | 1 | 0.60 | 209.45 | 1.00 | 0.97 | TRUE | TRUE |
| N15-503 | 221 | Signature_3_hc | 1 | 0.65 | 37.75 | 0.98 | 0.99 | TRUE | TRUE |
| N16-123 | 290 | Signature_17 | $< 10^{-3}$ | 0.51 | 147.51 | 1.00 | 0.05 | FALSE | FALSE |
| N16-145 | 254 | Signature_3_hc | 1 | 0.56 | 135.27 | 1.00 | 1.00 | TRUE | TRUE |
| N16-318 | 221 | Signature_3_lc | 1 | 0.67 | 56.87 | 1.00 | 0.89 | TRUE | FALSE |
| N16-60 | 260 | Signature_3_lc | $< 10^{-3}$ | 0.62 | 134.00 | 1.00 | 0.56 | TRUE | FALSE |
| N16-66 | 234 | Signature_clock | 1 | 0.60 | 18.18 | 0.83 | 0.16 | FALSE | FALSE |
| N17-106 | 268 | Signature_3_lc | 1 | 0.59 | 59.68 | 1.00 | 0.85 | TRUE | FALSE |
| N17-18 | 561 | Signature_3_lc | 1 | 0.58 | 299.69 | 1.00 | 0.68 | TRUE | FALSE |
| N17-188 | 229 | Signature_3_lc | 1 | 0.63 | 38.89 | 1.00 | 0.86 | TRUE | FALSE |
| N17-19 | 393 | Signature_17 | 1 | 0.39 | 283.21 | 1.00 | 0.03 | FALSE | FALSE |
| N17-204 | 463 | Signature_17 | $< 10^{-3}$ | 0.52 | 231.87 | 1.00 | 0.02 | FALSE | FALSE |
| N17-220 | 281 | Signature_3_lc | 1 | 0.71 | 41.65 | 0.97 | 0.89 | TRUE | FALSE |
| N17-221 | 1257 | Signature_3_lc | $< 10^{-3}$ | 0.34 | 187.50 | 1.00 | 0.21 | TRUE | FALSE |
| N17-233 | 320 | Signature_3_hc | 1 | 0.59 | 183.92 | 1.00 | 1.00 | TRUE | TRUE |
| N17-249 | 388 | Signature_3_hc | 1 | 0.70 | 217.35 | 1.00 | 1.00 | TRUE | TRUE |
| N17-26 | 587 | Signature_3_lc | 1 | 0.67 | 195.79 | 1.00 | 0.89 | TRUE | FALSE |
| N17-288 | 507 | Signature_3_lc | 1 | 0.73 | 0.00 | 0.50 | 0.50 | TRUE | FALSE |
| N17-334 | 399 | Signature_3_lc | 1 | 0.64 | 189.84 | 1.00 | 0.91 | TRUE | FALSE |
| N17-339 | 367 | Signature_3_hc | 1 | 0.67 | 78.43 | 1.00 | 1.00 | TRUE | TRUE |
| N17-369 | 795 | Signature_3_lc | 1 | 0.56 | 629.35 | 1.00 | 0.23 | TRUE | FALSE |
| N17-373 | 384 | Signature_3_lc | 1 | 0.70 | 143.40 | 1.00 | 0.92 | TRUE | FALSE |
| N17-384 | 714 | Signature_3_hc | 1 | 0.67 | 199.87 | 1.00 | 1.00 | TRUE | TRUE |
| N17-388 | 676 | Signature_3_lc | 1 | 0.67 | 285.75 | 1.00 | 0.57 | TRUE | FALSE |
| N17-425 | 351 | Signature_3_hc | 1 | 0.72 | 33.46 | 0.96 | 0.99 | TRUE | TRUE |
| N17-48 | 463 | Signature_3_lc | 1 | 0.57 | 325.29 | 1.00 | 0.64 | TRUE | FALSE |
| N18-142 | 326 | Signature_3_lc | 1 | 0.57 | 163.43 | 1.00 | 0.65 | TRUE | FALSE |
| N18-168 | 644 | Signature_3_hc | 1 | 0.68 | 312.39 | 1.00 | 0.99 | TRUE | TRUE |
| N18-215 | 395 | Signature_3_lc | 1 | 0.74 | 189.37 | 1.00 | 0.83 | TRUE | FALSE |
| N18-218 | 441 | Signature_3_lc | 1 | 0.68 | 129.87 | 1.00 | 0.92 | TRUE | FALSE |

| | | | | | | | | | |
|---|---|---|---|---|---|---|---|---|---|
| N18-258 | 380 | Signature_3_lc | 1 | 0.67 | 168.28 | 1.00 | 0.92 | TRUE | FALSE |
| N18-265 | 552 | Signature_17 | 1 | 0.51 | 323.33 | 1.00 | 0.13 | FALSE | FALSE |
| N18-267 | 604 | Signature_3_lc | $< 10^{-3}$ | 0.47 | 0.00 | 0.50 | 0.19 | TRUE | FALSE |
| N18-33 | 287 | Signature_3_lc | 1 | 0.64 | 198.31 | 1.00 | 0.91 | TRUE | FALSE |
| N18-50 | 338 | Signature_3_lc | 1 | 0.56 | 229.58 | 1.00 | 0.63 | TRUE | FALSE |
| N19-185 | 470 | Signature_3_lc | 1 | 0.74 | 268.23 | 1.00 | 0.92 | TRUE | FALSE |
| N19-212 | 935 | Signature_3_lc | 1 | 0.61 | 527.80 | 1.00 | 0.48 | TRUE | FALSE |
| N19-213 | 660 | Signature_17 | 1 | 0.49 | 377.09 | 1.00 | 0.03 | FALSE | FALSE |
| N19-26 | 540 | Signature_3_lc | 1 | 0.57 | 262.75 | 1.00 | 0.66 | TRUE | FALSE |
| N19-281 | 443 | Signature_3_lc | 1 | 0.62 | 307.74 | 1.00 | 0.84 | TRUE | FALSE |
| N19-310 | 840 | Signature_3_hc | 1 | 0.53 | 408.51 | 1.00 | 0.99 | TRUE | TRUE |
| N19-81 | 555 | Signature_3_lc | 1 | 0.77 | 211.66 | 1.00 | 0.93 | TRUE | FALSE |
| N19-94 | 492 | Signature_3_hc | 1 | 0.69 | 213.60 | 1.00 | 1.00 | TRUE | TRUE |

*total_snvs* indicates the number of SNVs in that sample

*categ* is the general category the sample falls into according to the dominant signatures. The Signature_3_hc indicates that the sample passes the strict threshold and hc stands for high confidence, and Signature_3_lc indicates that the sample passes the looser threshold but not the strict threshold.

*Signature_3_ml* is the total likelihood of clusters with Signature 3

*Signature_3_c* is the cosine similarity to Signature 3

*exp_sig3* is the exposure of Signature 3 calculated with NNLS

*Signature_3_l_rat* is the likelihood ratio of the NNLS decomposition with Signature 3 considering the possibility of an alternative decompositions without Signature 3. A value of 0.5 indicate that an NNLS decomposition without Signature 3 is as likely.

*Signature_3_mva* is the SigMA score indicating the presence of Signature 3 estimated by the gradient boosting classifier implemented in SigMA

*pass_mva* and *pass_mva_strict* are booleans indicating the presence of Signature 3 with the looser and strict selection *thresholds* that corresponds to 10% FPR and 1-5% FPR respectively

**Supplementary table 3 :** QC metrics

| Sample_id | Cellularity | Tumor sample | | | | Blood sample | | | |
|---|---|---|---|---|---|---|---|---|---|
| | | total | mean | median | % bases above 15 | total | mean | median | % bases above 15 |
| N17-106 | 70 | 85012753 | 50.24 | 33 | 58.7 | 30015646 | 17.74 | 13 | 46.2 |
| N19-94 | 60 | 37333393 | 22.06 | 12 | 44.5 | 45812799 | 27.07 | 19 | 53.6 |
| N17-309 | 55 | 66224299 | 39.13 | 28 | 57.9 | 46610457 | 27.54 | 20 | 53.9 |
| N19-81 | 60 | 62303841 | 36.82 | 28 | 58.3 | 48455935 | 28.63 | 19 | 54.2 |
| N17-221 | 75 | 47973052 | 28.35 | 20 | 53.8 | 53322028 | 31.51 | 23 | 56.1 |
| N18-258 | 80 | 51721196 | 30.56 | 26 | 58 | 53887342 | 31.84 | 29 | 59.4 |
| N17-388 | 80 | 79054713 | 46.72 | 36 | 59.7 | 57698084 | 34.1 | 27 | 57.8 |
| N18-265 | 100 | 60956847 | 36.02 | 26 | 58.5 | 58639455 | 34.65 | 25 | 58.4 |
| N18-215 | 70 | 66078269 | 39.05 | 27 | 59 | 60757357 | 35.9 | 32 | 60.4 |
| N18-168 | 80 | 56216309 | 33.22 | 23 | 56.3 | 61429852 | 36.3 | 28 | 58.1 |
| N18-218 | 60 | 82344691 | 48.66 | 36 | 60.6 | 61725710 | 36.48 | 32 | 60.1 |
| N17-334 | 33 | 47163975 | 27.87 | 28 | 59.4 | 64075472 | 37.87 | 27 | 58.5 |
| N17-19 | NA | 68874028 | 40.7 | 27 | 56.9 | 64669029 | 38.22 | 27 | 57 |
| N17-339 | 80 | 85198748 | 50.35 | 35 | 59 | 64988191 | 38.4 | 30 | 58.9 |
| N18-142 | 75 | 82065797 | 48.5 | 35 | 59.7 | 66919623 | 39.55 | 31 | 58.9 |
| N17-220 | 60 | 72600010 | 42.9 | 29 | 57.5 | 67773944 | 40.05 | 28 | 57.4 |
| N17-48 | 40 | 66988336 | 39.59 | 31 | 59.1 | 67975591 | 40.17 | 29 | 58.1 |
| N17-384 | 85 | 86315278 | 51.01 | 34 | 60 | 68585519 | 40.53 | 32 | 59.2 |
| N17-288 | 70 | 79859111 | 47.19 | 22 | 53.1 | 69012302 | 40.78 | 30 | 58.6 |
| N17-425 | 55 | 22407490 | 13.24 | 11 | 41.9 | 69921232 | 41.32 | 32 | 59.4 |
| N17-188 | 70 | 81039214 | 47.89 | 32 | 59 | 70917238 | 41.91 | 30 | 58.2 |
| N16-318 | NA | 124071025 | 73.32 | 37 | 57.8 | 72040915 | 42.57 | 24 | 55 |
| N17-204 | 55 | 88074932 | 52.05 | 37 | 59.8 | 73789292 | 43.61 | 31 | 58.5 |
| N18-50 | 70 | 64716064 | 38.24 | 30 | 58.3 | 75536089 | 44.64 | 34 | 59.7 |
| N17-373 | 50 | 110146290 | 65.09 | 48 | 61.3 | 76320591 | 45.1 | 35 | 59.7 |

| | | | | | | | | | |
|---|---|---|---|---|---|---|---|---|---|
| N15-346 | NA | 106868446 | 63.15 | 27 | 54.7 | 77811738 | 45.98 | 26 | 55.6 |
| N19-310 | 70 | 95319553 | 56.33 | 41 | 59.7 | 79033319 | 46.7 | 37 | 60.6 |
| N19-185 | 80 | 103059747 | 60.9 | 51 | 61.2 | 79306825 | 46.87 | 35 | 60.5 |
| N19-26 | 90 | 64651908 | 38.21 | 25 | 58.1 | 82054837 | 48.49 | 36 | 61 |
| N15-503 | NA | 75003233 | 44.32 | 24 | 54.7 | 82174207 | 48.56 | 28 | 55.8 |
| N18-33 | 75 | 91481306 | 54.06 | 38 | 60.4 | 83577385 | 49.39 | 37 | 59.9 |
| N17-18 | NA | 80773214 | 47.73 | 30 | 57.9 | 85196825 | 50.35 | 36 | 59.3 |
| N17-369 | 85 | 84538508 | 49.96 | 30 | 57.7 | 86799014 | 51.29 | 40 | 60.7 |
| N18-267 | 60 | 36542836 | 21.59 | 11 | 42.3 | 87985141 | 51.99 | 51 | 62.5 |
| N19-281 | 85 | 68366443 | 40.4 | 23 | 56.9 | 90420987 | 53.43 | 44 | 61.4 |
| N19-213 | 40 | 75573772 | 44.66 | 39 | 61.3 | 94117362 | 55.62 | 43 | 61.5 |
| N16-60 | NA | 97752762 | 57.77 | 23 | 53.6 | 97262786 | 57.48 | 29 | 55.6 |
| N17-233 | 50 | 83199792 | 49.17 | 35 | 59.5 | 100065834 | 59.13 | 43 | 60.8 |
| N19-212 | 80 | 83012728 | 49.06 | 40 | 61.4 | 102777849 | 60.74 | 47 | 62.1 |
| N16-66 | NA | 91252328 | 53.93 | 23 | 53.9 | 103711772 | 61.29 | 29 | 55.6 |
| N16-123 | NA | 92309475 | 54.55 | 25 | 54.7 | 105700413 | 62.46 | 29 | 55.8 |
| N17-26 | NA | 72789939 | 43.01 | 22 | 54.6 | 120854604 | 71.42 | 49 | 60.6 |
| N15-408 | NA | 109854457 | 64.92 | 32 | 56.4 | 122341336 | 72.3 | 37 | 57.3 |
| N16-145 | NA | 113626540 | 67.15 | 27 | 54.8 | 122505888 | 72.39 | 34 | 56.7 |
| N17-249 | 70 | 61920302 | 36.59 | 23 | 55.1 | 131674344 | 77.81 | 59 | 61.7 |
| N15-478 | NA | 80268724 | 47.43 | 22 | 53.8 | 159889099 | 94.49 | 45 | 58.2 |
| N19-62 | 35 | 62894472 | 37.17 | 23 | 57.7 | NA | NA | NA | NA |
| N18-242 | 40 | 60504576 | 35.75 | 30 | 59.2 | NA | NA | NA | NA |
| N19-278 | 40 | 86171685 | 50.92 | 40 | 60.8 | NA | NA | NA | NA |
| N18-308 | 50 | 52369558 | 30.95 | 26 | 58.1 | NA | NA | NA | NA |
| N17-296 | 65 | 77198218 | 45.62 | 27 | 56.6 | NA | NA | NA | NA |
| N17-149 | 70 | 89602336 | 52.95 | 32 | 58.3 | NA | NA | NA | NA |
| N17-251 | 70 | 77458926 | 45.77 | 31 | 58 | NA | NA | NA | NA |
| N17-398 | 70 | 78015784 | 46.1 | 34 | 59.6 | NA | NA | NA | NA |
| N17-412 | 70 | 87811039 | 51.89 | 33 | 58.4 | NA | NA | NA | NA |

| | | | | | | | | | |
|---|---|---|---|---|---|---|---|---|---|
| N18-235 | 70 | 77181523 | 45.61 | 29 | 59.4 | NA | NA | NA | NA |
| N18-281 | 70 | 28851304 | 17.05 | 15 | 49.5 | NA | NA | NA | NA |
| N18-365 | 70 | 38222520 | 22.59 | 19 | 54 | NA | NA | NA | NA |
| N19-291 | 70 | 81821625 | 48.35 | 35 | 60.2 | NA | NA | NA | NA |
| N17-402 | 75 | 77988701 | 46.09 | 33 | 59.6 | NA | NA | NA | NA |
| N18-119 | 75 | 39850725 | 23.55 | 15 | 49 | NA | NA | NA | NA |
| N18-174 | 75 | 72289117 | 42.72 | 32 | 58.9 | NA | NA | NA | NA |
| N17-214 | 80 | 63984666 | 37.81 | 25 | 56.6 | NA | NA | NA | NA |
| N18-139 | 80 | 94061473 | 55.59 | 28 | 55.3 | NA | NA | NA | NA |
| N18-373 | 80 | 52310939 | 30.91 | 11 | 43.4 | NA | NA | NA | NA |
| N15-170 | NA | 63575485 | 37.57 | 24 | 56 | NA | NA | NA | NA |
| N15-300 | NA | 55930057 | 33.05 | 15 | 49.2 | NA | NA | NA | NA |
| N15-308 | NA | 66315147 | 39.19 | 15 | 50 | NA | NA | NA | NA |
| N15-314 | NA | 71099920 | 42.02 | 18 | 51.7 | NA | NA | NA | NA |
| N15-337 | NA | 83470381 | 49.33 | 25 | 54.8 | NA | NA | NA | NA |
| N15-375 | NA | 117271912 | 69.3 | 26 | 54.1 | NA | NA | NA | NA |
| N15-399 | NA | 84228220 | 49.77 | 27 | 55.5 | NA | NA | NA | NA |
| N15-451 | NA | 104022821 | 61.47 | 32 | 56.5 | NA | NA | NA | NA |
| N15-465 | NA | 96282095 | 56.9 | 26 | 55 | NA | NA | NA | NA |
| N15-481 | NA | 95518893 | 56.45 | 29 | 56 | NA | NA | NA | NA |
| N15-486 | NA | 80131166 | 47.35 | 24 | 54.6 | NA | NA | NA | NA |
| N15-506 | NA | 66318201 | 39.19 | 19 | 52.5 | NA | NA | NA | NA |
| N15-76 | NA | 196240983 | 115.97 | 64 | 59.3 | NA | NA | NA | NA |
| N16-117 | NA | 98802368 | 58.39 | 27 | 55.2 | NA | NA | NA | NA |
| N16-160 | NA | 68044945 | 40.21 | 18 | 51.6 | NA | NA | NA | NA |
| N16-192 | NA | 72150248 | 42.64 | 23 | 54.8 | NA | NA | NA | NA |
| N16-26 | NA | 78520820 | 46.4 | 25 | 55.2 | NA | NA | NA | NA |
| N16-260 | NA | 102251440 | 60.42 | 25 | 54.3 | NA | NA | NA | NA |
| N16-71 | NA | 110516602 | 65.31 | 34 | 57.3 | NA | NA | NA | NA |
| N17-05 | NA | 86652689 | 51.21 | 15 | 49.4 | NA | NA | NA | NA |

| | | | | | | | | | |
|---|---|---|---|---|---|---|---|---|---|
| N18-156 | NA | 109403037 | 64.65 | 38 | 58.4 | NA | NA | NA | NA |

QC metrics for each sample. including histopathological cellularity assessment (Cellularity) and coverage information (Total number of reads, mean and median coverage, and percentage of bases with coverage above 15 X).
