# Supplementary material for: Efficacy of platinum-based chemotherapy in metastatic breast cancer and HRD biomarkers: utility of exome sequencing
Source: NPJ Breast Cancer. 2022 Mar 4;8:28. doi: 10.1038/s41523-022-00395-0 (PMC8897409; doi:10.1038/s41523-022-00395-0)
Supplement: Supplementary file 2 — Reporting Summary [file 41523_2022_395_MOESM2_ESM.pdf]

nature portfolio

Corresponding author(s): LADOIRE SYLVAIN - sladoire@cgfl.fr

Last updated by author(s): Jan 22, 2022

Reporting Summary

Nature Portfolio wishes to improve the reproducibility of the work that we publish. This form provides structure for consistency and transparency in reporting. For further information on Nature Portfolio policies, see our Editorial Policies and the Editorial Policy Checklist.

Statistics

For all statistical analyses, confirm that the following items are present in the figure legend, table legend, main text, or Methods section.

n/a	Confirmed	
☐	☒	The exact sample size (*n*) for each experimental group/condition, given as a discrete number and unit of measurement
☒	☐	A statement on whether measurements were taken from distinct samples or whether the same sample was measured repeatedly
☐	☒	The statistical test(s) used AND whether they are one- or two-sided<br>*Only common tests should be described solely by name; describe more complex techniques in the Methods section.*
☐	☒	A description of all covariates tested
☒	☐	A description of any assumptions or corrections, such as tests of normality and adjustment for multiple comparisons
☐	☒	A full description of the statistical parameters including central tendency (e.g. means) or other basic estimates (e.g. regression coefficient) AND variation (e.g. standard deviation) or associated estimates of uncertainty (e.g. confidence intervals)
☐	☒	For null hypothesis testing, the test statistic (e.g. *F*, *t*, *r*) with confidence intervals, effect sizes, degrees of freedom and *P* value noted<br>*Give P values as exact values whenever suitable.*
☒	☐	For Bayesian analysis, information on the choice of priors and Markov chain Monte Carlo settings
☒	☐	For hierarchical and complex designs, identification of the appropriate level for tests and full reporting of outcomes
☒	☐	Estimates of effect sizes (e.g. Cohen's *d*, Pearson's *r*), indicating how they were calculated

Our web collection on statistics for biologists contains articles on many of the points above.

Software and code

Policy information about availability of computer code

Data collection	No software was used.
Data analysis	Burrows–Wheeler aligner (BWA v.0.7.15), Picard (v.2.5), VarScan (v2.4.3), Mutect (v1.1.7), Strelka (v2.9.2), DeconstructSigs (v1.8.0), GraphPad Prism (v7.03), R (v4.0.2)

For manuscripts utilizing custom algorithms or software that are central to the research but not yet described in published literature, software must be made available to editors and reviewers. We strongly encourage code deposition in a community repository (e.g. GitHub). See the Nature Portfolio guidelines for submitting code & software for further information.

Data

Policy information about availability of data

All manuscripts must include a data availability statement. This statement should provide the following information, where applicable:

- Accession codes, unique identifiers, or web links for publicly available datasets
- A description of any restrictions on data availability
- For clinical datasets or third party data, please ensure that the statement adheres to our policy

The datasets generated and analyzed during the current study are available on reasonable request.

Field-specific reporting

Please select the one below that is the best fit for your research. If you are not sure, read the appropriate sections before making your selection.

☒ Life sciences ☐ Behavioural & social sciences ☐ Ecological, evolutionary & environmental sciences

For a reference copy of the document with all sections, see nature.com/documents/nr-reporting-summary-flat.pdf

Life sciences study design

All studies must disclose on these points even when the disclosure is negative.

Sample size	The analysis were performed with 86 patients.
Data exclusions	No data exclusions
Replication	Not concerned
Randomization	No randomization was made in our study
Blinding	No group and no blinding

Reporting for specific materials, systems and methods

We require information from authors about some types of materials, experimental systems and methods used in many studies. Here, indicate whether each material, system or method listed is relevant to your study. If you are not sure if a list item applies to your research, read the appropriate section before selecting a response.

Materials & experimental systems

n/a	Involved in the study	
☒	☐	Antibodies
☒	☐	Eukaryotic cell lines
☒	☐	Palaeontology and archaeology
☒	☐	Animals and other organisms
☐	☒	Human research participants
☐	☒	Clinical data
☒	☐	Dual use research of concern

Methods

n/a	Involved in the study	
☒	☐	ChIP-seq
☒	☐	Flow cytometry
☒	☐	MRI-based neuroimaging

Human research participants

Policy information about studies involving human research participants

Population characteristics	Exclusively women, with a metastatic breast cancer, all with tumor exome sequencing
Recruitment	Eighty-six patients with MBC in whom WES (Whole Exome Sequencing) analysis was performed and interpreted according to the Molecular Tumor Board of the Georges François Leclerc Cancer Center were included in this single-centre, retrospective study. Patients were enrolled between June 2018 and March 2020, and all received at least one line of platinum-based chemotherapy.
Ethics oversight	The study was approved by the CNIL (French national commission for data privacy) and the local ethics committee, and was performed in accordance with the Helsinki Declaration and European legislation.

Note that full information on the approval of the study protocol must also be provided in the manuscript.

Clinical data

Policy information about clinical studies

All manuscripts should comply with the ICMJE guidelines for publication of clinical research and a completed CONSORT checklist must be included with all submissions.

Clinical trial registration	Not concerned - no clinical trial registration number
Study protocol	Our sutdy protocol is available in the part 'Materials and Methods" of our protocol
Data collection	Retrospective data collection, on medical records

Outcomes

Survival analysis was performed using the survival R library.
PFS1 was defined by the European Medicines Agency (EMA) as the time since the start of the last treatment prior to progression, as defined by RECIST 1.1 or clinical progression. PFS2 was defined as the time from the start of platinum therapy to progression, as defined by RECIST 1.1, clinical progression, or death from any cause. PFS2 was compared with PFS1 for each patient using the PFS2/PFS1 ratio (or growth modulation index 40). Overall survival (OS) was defined as the time from the start of platinum therapy to death from any cause.
Radiological response to platinum-based chemotherapy was determined locally, and assessed by clinical and radiological evaluations every 2-4 months for each patient.
